# Antiproliferative and Pro-Apoptotic Activity and Tubulin Dynamics Modulation of 1*H*-Benzimidazol-2-yl Hydrazones in Human Breast Cancer Cell Line MDA-MB-231

**DOI:** 10.3390/molecules29102400

**Published:** 2024-05-20

**Authors:** Denitsa Yancheva, Maria Argirova, Irina Georgieva, Vanya Milanova, Maya Guncheva, Miroslav Rangelov, Nadezhda Todorova, Rumiana Tzoneva

**Affiliations:** 1 Institute of Organic Chemistry with Centre of Phytochemistry, Bulgarian Academy of Sciences, Acad. G. Bonchev St., Build. 9, 1113 Sofia, Bulgaria; maria.argirova@orgchm.bas.bg (M.A.); maya.guncheva@orgchm.bas.bg (M.G.); miroslav.rangelov@orgchm.bas.bg (M.R.); 2 Institute of Biophysics and Biomedical Engineering, Bulgarian Academy of Sciences, Acad. G. Bonchev St., Build. 21, 1113 Sofia, Bulgaria; igeorgieva@biomed.bas.bg (I.G.); vanyamilanova99@gmail.com (V.M.); 3 Institute of Biodiversity and Ecosystem Research, Bulgarian Academy of Sciences, 2 Gagarin Str., 1113 Sofia, Bulgaria; nadeshda@abv.bg

**Keywords:** benzimidazol-2-yl hydrazones, tubulin polymerization, mitotic blockage, cytotoxicity, MDA-MB-231 cells

## Abstract

(1) Background: The aim of the work is the evaluation of in vitro antiproliferative and pro-apoptotic activity of four benzimidazole derivatives containing colchicine-like and catechol-like moieties with methyl group substitution in the benzimidazole ring against highly invasive breast cancer cell line MDA-MB-231 and their related impairment of tubulin dynamics. (2) Methods: The antiproliferative activity was assessed with the MTT assay. Alterations in tubulin polymerization were evaluated with an in vitro tubulin polymerization assay and a docking analysis. (3) Results: All derivatives showed time-dependent cytotoxicity with IC_50_ varying from 40 to 60 μM after 48 h and between 13 and 20 μM after 72 h. Immunofluorescent and DAPI staining revealed the pro-apoptotic potential of benzimidazole derivatives and their effect on tubulin dynamics in living cells. Compound **5d** prevented tubulin aggregation and blocked mitosis, highlighting the importance of the methyl group and the colchicine-like fragment. (4) Conclusions: The benzimidazole derivatives demonstrated moderate cytotoxicity towards MDA-MB-231 by retarding the initial phase of tubulin polymerization. The derivative **5d** containing a colchicine-like moiety and methyl group substitution in the benzimidazole ring showed potential as an antiproliferative agent and microtubule destabilizer by facilitating faster microtubule aggregation and disrupting cellular and nuclear integrity.

## 1. Introduction

Microtubules are key components of the cytoskeleton. They are composed of α-tubulin and β-tubulin heterodimers, forming a long, filamentous, tube-shaped supramolecular complex with a 25 nm diameter. Since they are intrinsically polarized, they play a role in the regulation and maintenance of cell polarity [1,2,3]. During the interphase, they span radially, providing tracks for the fast transport of cargoes by members of two classes of molecular motor proteins—dynein and kinesin [4]. During mitosis, microtubules (MTs) rearrange to form the mitotic spindle, composed of MTs stretching from two opposing spindle poles: the minus ends, anchored to the poles, and the plus ends, extending away from the poles and binding to chromosomes [5]. This arrangement is responsible for the correct alignment and segregation of the chromosomes. Therefore, in this way, the stages of mitosis that cells go through during normal cell division can be studied closely.

Microtubule-targeting agents (MTAs) are a highly successful class of cancer drugs with therapeutic applications in both hematopoietic and solid tumors [6]. They exert their antitumor activity by disrupting the microtubules, resulting in the induction of cell cycle arrest in the G2-M phase due to suppression of spindle-microtubule dynamics. As a result, chromosome segregation is interrupted, and this could trigger the slowing down or blocking of mitosis at the metaphase–anaphase transition and induction of apoptotic cell death [7]. At higher concentrations, antimitotic drugs work through one of the following mechanisms: they either inhibit the polymerization of microtubules, leading to destabilization and a reduction in polymer mass, which blocks cell proliferation at metaphase during mitosis, or they promote the polymerization of microtubules, stabilizing them and increasing the polymer mass [3,8,9,10]. Based on their mechanism of action, antimitotic agents can be split into two main groups: (1) microtubule-destabilizing agents (MDAs), which inhibit microtubule polymerization at high concentrations and include several compounds such as vinca alkaloids (vinblastine, vincristine, etc.) and colchicine, and (2) microtubule-stabilizing agents (MSAs), which stimulate microtubule polymerization, such as taxanes (paclitaxel and docetaxel) [3,11,12].

**Figure 1:**
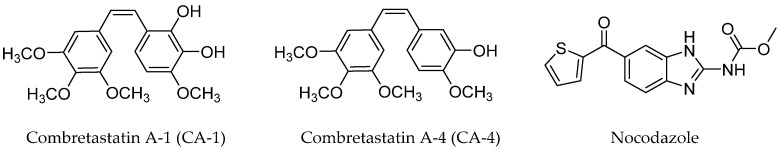
MTAs targeting colchicine binding site on β-tubulin.

The main groups can be further divided into subgroups based on their targeting of different binding sites on the αβ-tubulin dimer. Currently, at least eight binding sites are recognized in the αβ-tubulin dimer, most of them being located on β-tubulin [13,14]. Research efforts have primarily focused on exploring the binding sites of vinca alkaloids, colchicine and taxanes. The vinca domain is located at the inter-dimer interface between two longitudinally aligned tubulin dimers. The taxane site is found in a deep hydrophobic pocket at the lateral interface in the microtubule lumen between adjacent protofilaments. Finally, the colchicine site is located at the intra-dimer interface between β-tubulin and α-tubulin. Vinca alkaloids, taxanes and their derivatives have been well studied, and some are applied in chemotherapy for different types of cancers [11,12,15]. The natural products combretastatin A-1 (CA-1) and A-4 (CA-4) and the synthetic compound nocodazole (Figure 1) are the most promising drugs for cancer treatment among the versatile antimitotic drugs that bind to the colchicine site.

CA-1 and CA-4 are stilbene compounds bearing a trimethoxypenyl moiety combined with catechol or a vanillin-derived fragment [16]. The water-soluble prodrug of CA-1, a dipotassium diphosphate derivative (OXi4503), is currently in clinical trials for patients with relapsed or refractory acute myeloid leukemia [17]. Fosbretabulin (Zybrestat), a water-soluble version of CA-4 designed for better absorption, and Ombrabulin, a variant of CA-4 modified with serine, are both undergoing clinical trials of their effectiveness as independent cancer treatments as well as in combination with other chemotherapeutics or radiation therapy [18,19]. Nocodazole, a member of the benzimidazole family, attracted attention due to its potent tubulin inhibition, significant antiproliferative activity and wide use in clinical research [12]. Over the years, several structural modifications of these drugs, which improved their antitumor activity, have been reported [15,20,21]. Despite the great number of synthesized compounds targeting the colchicine binding site, no colchicine site inhibitors have been approved for cancer therapy yet.

The diverse representatives of the benzimidazole family exhibiting potent tubulin polymerization, such as denibulin, NSC:761109/1 and 2-aryl-benzimidazoles derivatives of dehydroabietic acid, outline the benzimidazole nucleus as a useful pharmacophore for the design of novel tubulin polymerization inhibitors [22,23,24,25,26].

We have reported recently the synthesis of a library of 1*H*-benzimidazol-2-yl hydrazones containing hydroxy, methoxy and fluoro groups in the phenyl moiety or a 1,3-benzodioxolyl substituent [27,28]. These compounds exhibited excellent in vitro anthelmintic activity against isolated Trichinella spiralis muscle larvae, as well as a cytotoxic effect on the human malignant cell lines MCF-7 and AR-230 and normal fibroblast cell lines 3T3 and CCL-1 [27,28,29]. They also demonstrated radical-scavenging activity against stable free radicals DPPH and ABTS and biologically relevant peroxyl radicals, a significant protective effect in model systems with deoxyribose and lecithin and metal-chelating activity. The interaction with tubulin was explored through molecular docking and an in vitro tubulin polymerization assay that revealed that most compounds prolong the nucleation phase and retard the tubulin polymerization in a manner comparable to nocodazole.

**Scheme 1:**
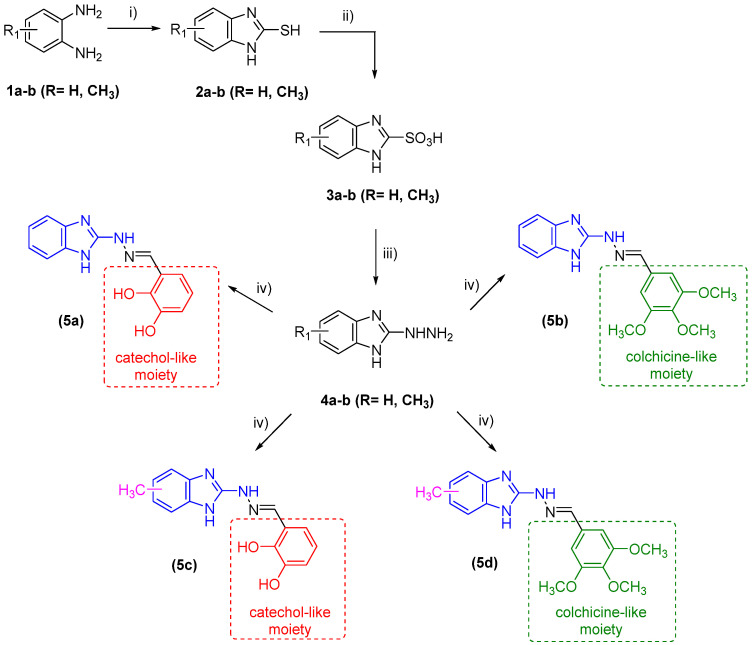
Reaction pathway for the synthesis of 1*H*-benzimidazol-2-yl hydrazones **5a**–**d**: (i) CS2, KOH, EtOH, refluxing, 50% acetic acid; (ii) oxidation in a 50% ethanol solution of potassium permanganate, 1 h, acidification by hydrochloric acid to pH = 1; (iii) refluxing in excess of 99% hydrazine hydrate, 3 h; (iv) condensation with substituted benzaldehyde in 99% ethanol, molar ratio 1:1, 3–4 h.

In this study, we expanded our evaluation of 1*H*-benzimidazol-2-yl hydrazones by testing their potential antiproliferative and pro-apoptotic activities against the highly invasive breast cancer cell line MDA-MB-231 and related impairment of cancer cells’ tubulin dynamics. We focused on two of the most promising compounds from the 1*H*-benzimidazol-2-yl hydrazone series (**5a** and **5b**, Scheme 1) and two newly synthesized derivatives with a methyl group in the benzimidazole ring (**5c** and **5d**, Scheme 1). The impact of 1*H*-benzimidazol-2-yl hydrazones on tubulin polymerization was further evaluated in vitro using purified porcine tubulin. This allowed us to ascertain the effect of the methyl substitution in the benzimidazole ring and to compare it with the microtubule-modulating agents paclitaxel and nocodazole. Additionally, the interactions of the studied compounds with tubulin were elucidated by molecular docking in the colchicine binding site. 

## 2. Results and Discussion

### 2.1. Synthesis of Target Compounds

The reaction pathway for the synthesis of the target benzimidazolyl hydrazones is presented in Scheme 1 in accordance with the previously described procedure [27,28]. Briefly, o-phenylenediamine was used to obtain the benzimidazole thiols **2a**,**b**, which then reacted with potassium permanganate to afford benzimidazolyl sulfonic acids **3a**,**b**. The 1H-benzimidazol-2-yl hydrazines **4a**,**b** were obtained by nucleophilic substitution with hydrazine hydrate. In the final step, **4a**,**b** reacted with 2,3-hydroxybenzaldehyde or 3,4,5-trimethoxybenzaldehyde, and four 1H-benzimidazol-2-yl hydrazones **5a**–**d** were obtained where the substitution in the benzimidazole ring varied (a methyl group vs. nonsubstituted ring) along with the phenyl part (catechol-like vs. colchicine-like moiety) (Scheme 1).

**Scheme 2:**
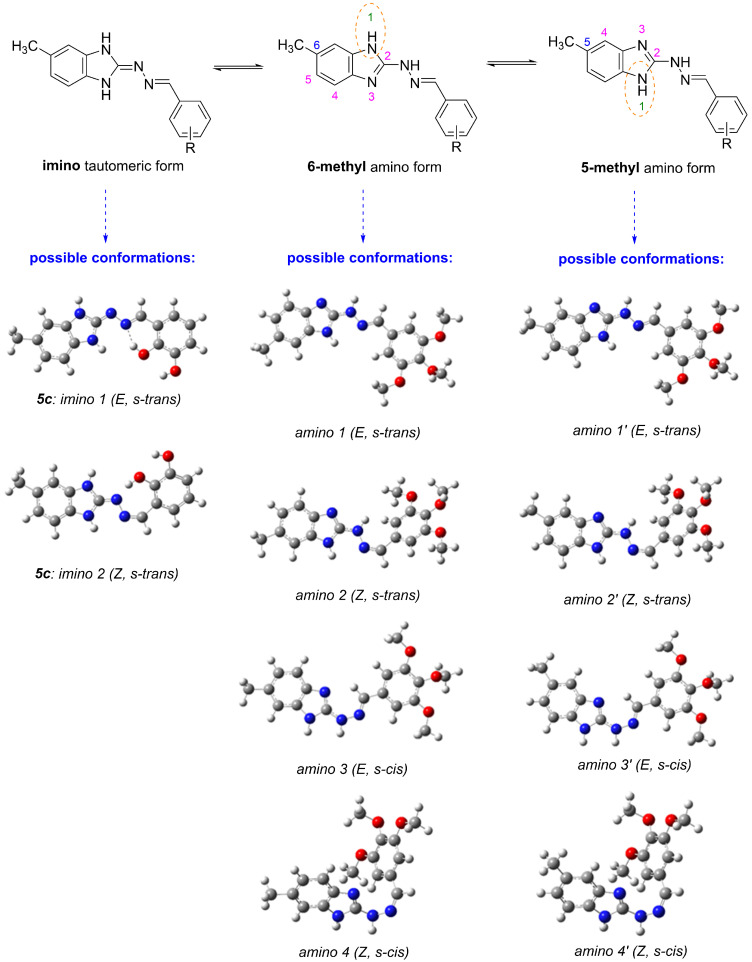
Tautomeric equilibria and possible conformers for 1H-benzimidazolyl hydrazones illustrated by the optimized geometry of compounds **5c** and **5d**.

Considering the substituent in the benzimidazole fragment, the hydrogen atom in the amino group of the benzimidazole nucleus could migrate from N1 to N3, resulting in two possible positional isomers, 5-methyl and 6-methyl, as well as two tautomeric forms, imino and amino (Scheme 2). For each tautomer and respective positional isomer (5- and 6-isomer), there are several possible conformations arising from the rearrangement around the double azomethine (C=N) and the single N-N bond—E,s-trans, Z,s-trans, E,s-cis and Z,s-cis (Scheme 2). The possible isomers were studied using DFT calculations at the B3LYP/6-311++G(d, p) level of theory in the gas phase, water and DMSO solution. Tables S1–S6 in the Supplementary Materials summarize the total Gibbs free energies and relative (ΔG) energies.

In a nonpolar medium (gas phase), the energetically most favorable structure of compound **5c** is the imino form in E,s-trans conformation (imino 1 in Scheme 2) followed by the Z,s-trans imino form (imino 2 in Scheme 2). The energy difference between the two conformers is 5.43 kJ·mol^−1^, corresponding to the Boltzmann population 86.42:9.67% (Table S3). The amino forms of **5c** (either as 6-isomer or 5-isomer) are less energetically favored (Table S3) and are expected to be present below 4% in the gas phase. However, they gain importance in the water phase where the three energetically most favored structures are imino 1 (E,s-trans), amino 1 (6-isomer in E,s-trans conformation) and amino 1′ (5-isomer in E,s-trans conformation) with the predicted Boltzmann ratio of 79.97:8.63:6.75%, respectively. In DMSO solvent, the energy differences between the three forms are even lower, so they are expected to coexist in solution with a ratio of 53.25:21.40:25.35% (Table S3). 

For compound **5d** in the gas phase, the two amino forms in E,s-trans conformation (amino 1 and amino 1′ in Scheme 2) are significantly more stable than the imino forms. The energy difference between amino 1 and amino 1′ is insignificant (0.01 kJ·mol*^−^*^1^), resulting in an almost equal Boltzmann ratio—51.10:49.90% (Table S6). In the water phase, the DFT calculations predicted that the amino 1′ form would prevail over the amino 1 form with a ratio of 69.31:30.69%, respectively, while in DMSO, amino 1 would be dominant (Boltzmann ratio for amino 1/amino 1′ = 59.95:40.05%, Table S6). In summary, both positional isomers of **5d** are expected to coexist as E,s-trans in water and DMSO solution.

The synthesis of the two 1H-benzimidazol-2-yl hydrazones, **5a** and **5b**, that bear the unsubstituted benzimidazole ring is described in more detail in our previous publication along with the spectral data confirming their structure [28]. The structures of the newly obtained 1H-benzimidazol-2-yl hydrazones, **5c** and **5d**, were established by HRMS, FT-IR, ^1^H-NMR and ^13^C-NMR spectroscopy, and the data are provided in the experimental section. One of the most characteristic bands in the IR spectra is that of the formation of the azomethine bond (C=N) at ca. 1620 cm*^−^*^1^. The N-H group of the benzimidazole ring and that of the hydrazine chain are characterized by the presence of a peak in the region 3370–3250 cm*^−^*^1^. The hydrazone **5c**, which contains hydroxyl groups at ortho- and meta positions, showed a band for C–O stretching vibrations around 1260 cm*^−^*^1^. In the IR spectra of compound **5d,** bands for the C–O stretching vibrations of the methoxyl groups were registered within the interval 1270–1050 cm*^−^*^1^ with moderate to strong intensity. 

In the ^1^H-NMR spectra of the target hydrazones, singlets at ca. 8 ppm were registered, thus confirming the formation of the azomethine bond. The benzimidazole protons were found as multiplets in the region 6.9–7.3 ppm, while those for the phenyl moiety were found around 7–7.8 ppm. At 11.35 ppm and 9.35 ppm, two broad signals were observed for the protons for the amino and hydroxyl groups. The chemical shifts for the OCH_3_ groups varied in the range of 3.9–3.7 ppm. The substitution of the benzimidazole ring with a methyl group gave rise to a singlet at 2.32–2.40 ppm. In the ^13^C-NMR spectra, the signals for the azomethyne C atoms were found at ca. 141–143 ppm. C atoms bonded to the hydroxy and methoxy groups in the phenyl rings resonate at ca. 145 ppm and 153 ppm, respectively (Figures S3 and S6). The signals for the C atoms neighboring the nitrogen atoms from the benzimidazole ring were observed within the interval 131–145 ppm. The rest of the signals for aromatic C atoms were found within the range 100–125 ppm. The C atoms from the methoxy groups resonate at ca. 56 ppm, while those for the methyl group in the benzimidazole cycle were registered at ca. 21 ppm. In order to support the assignments of the NMR signals, ^1^H- and ^13^C-NMR chemical shifts of **5c** and **5d** (in the expected imino and amino forms in DMSO solution) were calculated by using the GIAO method. The obtained values are presented in Tables S7 and S8. As can be seen there, several C atoms are predicted with very close chemical shifts. In agreement with this, the signals detected in the NMR spectra are less than the total number of C atoms in **5c** and **5d**. Although these compounds are expected to be present as a mixture of three and two tautomeric/isomeric forms, respectively, no doubling of the experimental signals was observed in the ^1^H- or the ^13^C-NMR spectra. This could be explained by the small energy difference between the tautomeric/isomeric forms (less than 3 kJ·mol*^−^*^1^), as estimated by the DFT calculations, and their easy interconversion.

### 2.2. In Vitro Effect on Tubulin Polymerization and Docking Study of Tubulin–Ligand Interactions

**Figure 2:**
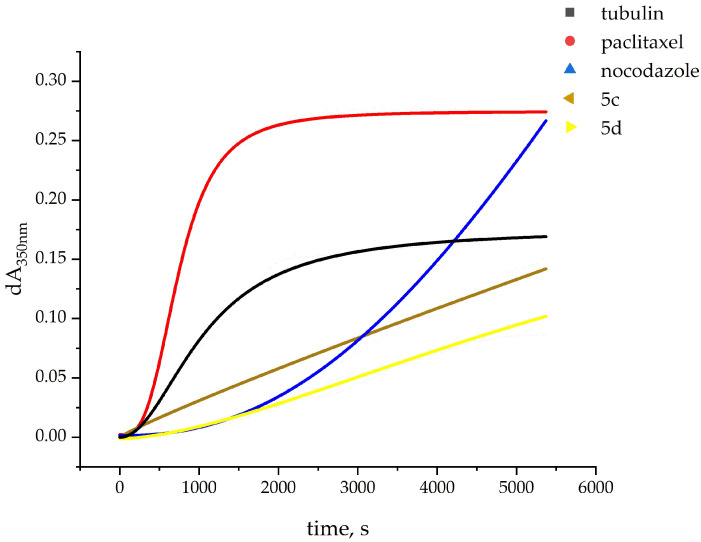
Tubulin (final 54 µM) in the presence of polymerization buffer containing 1 mM guanosine triphosphate (PB-GTP buffer), 10 µM paclitaxel, 10 µM nocodazole and 10 µM of compounds **5c** and **5d**. The reaction was conducted in a thermostated spectrophotometric chamber at 37 °C for 90 min. Turbidity was measured at 350 nm every 30 s.

The effect of 1*H*-benzimidazol-2-yl hydrazones **5a**–**d** on tubulin polymerization was evaluated in vitro on purified porcine tubulin (Figure 2). The tubulin polymerization assay was carried out at a 10 µM concentration of the nocodazole and paclitaxel reference drugs and compounds **5a**–**d**.

The in vitro tubulin polymerization assay showed that the spontaneous tubulin polymerization proceeded at the initial rate of 75.3 a.u.s^−1^.10^−6^, while nocodazole delayed the polymerization start by 935 s and lowered the initial rate to 52 a.u.s^−1^.10^−6^ (Table 1). In contrast, paclitaxel accelerated the polymerization to 167 a.u.s^−1^.10^−6^. As reported earlier, the two hydrazones without a substituent in the benzimidazole ring **5a** and **5b** are characterized by 1461 and 988 s lag time, respectively, and considerable reduction in the initial rate—13.4 for **5a** and 20.6 a.u.s^−1^.10^−6^ for **5b** [29]. The introduction of a methyl group in the benzimidazole ring induced a slightly different effect on the tubulin polymerization. While **5c** (with catechol-like moiety) showed no lag time and an initial rate of polymerization of 27 a.u.s^−1^.10^−6^, **5d** (with colchicine-like moiety) delayed the polymerization by 600 s, followed by a polymerization phase with a 12.9 a.u.s^−1^.10^−6^ rate. Compound **5d**, with a methyl group in the benzimidazole ring and a colchicine-like fragment in the hydrazone chain, exhibited the most significant reduction in the tubulin polymerization rate among the tested compounds. A comparison of the tubulin polymerization curves in the presence of all four compounds **5a**–**d**, paclitaxel and nocodazole is given in the Supplementary Materials (Figure S7).

**Figure 3:**
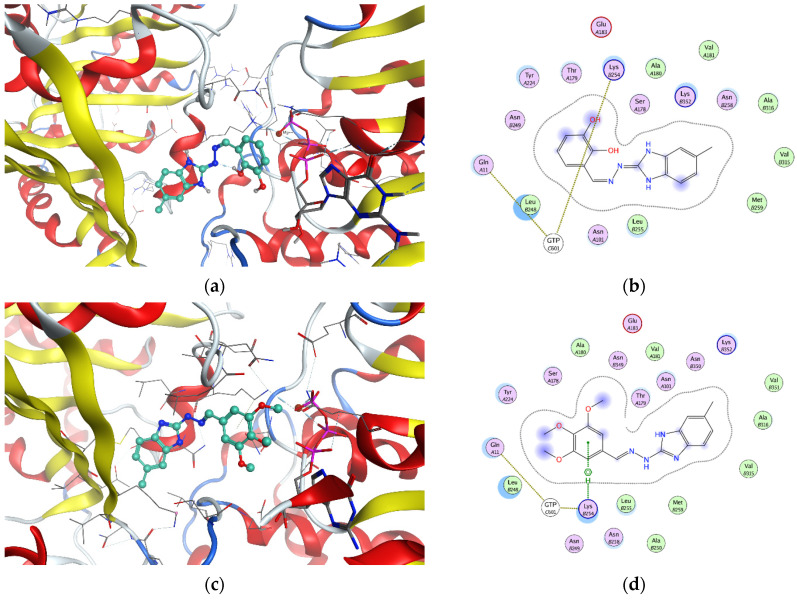
Interactions of ligands **5a**–**d** at the colchicine binding site of the tubulin dimer: three-dimensional representation of the ligands poses (left (**a**,**c**)) and interaction maps (right (**b**,**d**)); the following colors are used to depict the different components: proximity contour—a black dotted line; polar amino acids—a pink line; lipophilic amino acids—a green line; basic amino acids—a blue line; acidic amino acids—a red line; hydrogen bond interactions—blue arrows; lipophilic interactions—green dotted lines. Docked ligands **5a**–**d** are shown by balls and sticks; GTP is shown by sticks.

A molecular docking study was conducted to provide a better insight into the possible binding modes of the 1*H*-benzimidazol-2-yl hydrazones with tubulin using the MOE program package [30]. The interactions of the ligands **5a**–**d** were modeled based on the X-ray structure of the colchicine complex of αβ-tubulin with colchicine and vinblastine, PDB ID: 1Z2B [31]. Previous studies have shown that the unsubstituted benzimidazole ring of ligands **5a** and **5b** is oriented in the active pocket towards the hydrophobic amino acid residues of β-tubulin and the phenyl moiety (either catechol-like or colchicine-like) and faces the polar amino acid residues of α-tubulin. In the case of methyl-substituted ligands, **5c** and **5d**, the phenyl rings lie again in close proximity to the GTP and some of the key amino acid residues that are believed to be important for the interactions preventing the “curved-to-straight” conformational change during the tubulin assembly—the polar amino acid residues Ser178 and Thr179 of the α-tubulin T5 loop, Gln11 in the T1 loop of α-tubulin or Lys254 within the H8 helix of β-tubulin [25,32,33]. The interaction maps of ligands **5c-d** (Figure 3) indicated that the binding of **5c** and **5d** is based mainly on van der Waals interactions, and in the case of **5d**, binding is also based on some lipophilic interactions between the trimethoxyphenyl ring and Lys254 from the β-tubulin unit, positioned in close proximity to the GTP, are formed in addition.

Given the broad spectrum of targets associated with benzimidazole derivatives, including but not limited to Topoisomerase II, VEGFR and EGFR [34], it is plausible to consider that tubulin may not be the exclusive target for the compounds in question. For example, research by Husain et al. demonstrated that (E)-5-(1*H*-benzo[d]imidazol-2-yl)-3-(3,4,5-trimethoxybenzylidene)furan-2(3H)-one, despite its imperfect alignment within the VEGFR binding site compared to its counterparts, showcased superior cytotoxicity [35]. Although the exploration of additional binding interactions falls outside the scope of this study, there is evidence by various researchers of similar cytotoxic behaviors among benzimidazole derivatives with and without the addition of a methyl group at the 5(6) position of the benzimidazole core. For instance, a selenediazole benzimidazole derivative with a methyl group displayed heightened antiproliferative effects compared to its unmethylated counterpart in the MDA-MB-231 cell line [36]. This compound also surpassed others in the same group that featured –Cl or –Br substituents in triggering G2/M cell cycle arrest, apoptosis and a reduction in ERK expression and phosphorylation. A similar pattern of cytotoxicity was noted in benzimidazole-based Schiff bases with varying aromatic rings and substituents in the benzimidazole core [37] and in terphenyl benzimidazoles. Interestingly, research by Kamal et al. [38] revealed that substituting a methyl group with an OH group provided comparable antiproliferative effects but enhanced G2/M arrest and inhibition of tubulin polymerization. Further studies by the same team comparing the impact of a fluorine atom with a methyl group on the benzimidazole nucleus within benzimidazole–perimidine hybrids once again demonstrated superior efficacy in apoptosis induction, mitochondrial potential alterations and ROS production with the methylated compound. There were similar findings in a series of tetracyclic benzimidazole derivatives and tetracyclic and pentacyclic perimidine derivatives featuring one or two methyl groups in the benzimidazole core [39]. In other alkylsulfonyl benzimidazole compounds, the antiproliferative activity is further enhanced by the introduction of a methyl group into the sulfonyl group, with values ranging from 1 to 40 μM. Molecular docking studies suggest that the presence of halogen atoms or sulfonyl groups is important for stabilizing the complex with DNA through hydrogen bonds [40] Hence, the incorporation of a methyl group in the benzimidazole nucleus seems to enhance the antiproliferative properties of benzimidazole derivatives, potentially due to opportunistic binding of individual compounds to cellular targets that share similar binding sites. Another strategy for elevation of both cytotoxicity and antibacterial efficacy is the introduction of electron acceptor substituents such as a cyano group or chlorine atom [37,41].

### 2.3. Determining Cell Viability by MTT Assay

To determine cell viability after treatment with 1*H*-benzimidazol-2-yl hydrazones **5a**–**d**, an MTT test was performed, and IC_50_ values were calculated as described in Materials and Methods. Based on our results, some interesting structure–activity relationships (SARs) could be derived. As shown in Table 2, cell viability was not altered significantly when using compounds with a catechol-like moiety (**5a**) or colchicine-like moiety (**5b**) after 48 h of treatment, while after a 72 h treatment, the overall cytotoxicity increased, and IC_50_ for **5a** became lower than that for **5b**. When looking at the above structures with additional substitutions with a methyl group in the 5(6)-position of the benzimidazole heterocycle, namely **5c** and **5d**, we can assume that the cell viability retained the same values as for their basic structures (without CH_3_ group substitution) after 48 h of incubation (Table 2). The only significant difference in cell viability between both compounds with a colchicine-like moiety can be observed after 72 h (Table 2). According to Table 2, the compound with an additional CH_3_ group (**5d**) exhibited stronger cytotoxicity than **5b**. On the whole, the antiproliferative effect of all compounds is more prominent after 72 h of treatment. Obviously, the presence of a methyl functional group at the 5(6)-position of the benzimidazole ring combined with the substitution with three methoxy groups at the R3, R4 and R5 positions of the phenyl ring (forming a colchicine-like moiety) proved the best antiproliferative derivatives against the triple-negative breast cancer cell line MDA-MB-231 (Table 2).

Interestingly, compound **5b,** which exhibited the lowest cytotoxicity against the triple-negative breast cancer cell line MDA-MB-231, showed the most remarkable cytotoxicity (1.2 ± 0.2 µM) for the hormone-dependent MCF-7 cell line [29]. This could be explained by the more subtle mitotic arrest in MDA-MB-231 cells than in MCF-7 cells treated with anticancer drugs [42]. However, we proved that incorporating the drug into fluorescent micellar carriers improves its cytotoxicity against MDA-MB-231 approximately four-fold [43].

**Table 2:** Calculated IC_50_ for each compound 48 and 72 h post-incubation with MDA-MB-231.

Compound	IC_50_ (µM)	
	48 h	72 h
Unsubstituted Benzimidazole Ring		
**5a**	43.93 ± 9.3	16.04 ± 2.2
**5b**	60.08 ± 15.3	20.30 ± 4.3 ^1^
5(6)-Methyl Benzimidazole Ring		
**5c**	47.28 ± 8.0	14.83 ± 3.8
**5d**	40.3 ± 4.1	12.65 ± 2.5
Reference Compound		
Nocodazole	0.38 ^2^	

^1^ [43]; ^2^ IC_50_ for 24 h [23].

### 2.4. Investigation of Microtubule Organization and Nuclear Morphology

**Figure 4:**
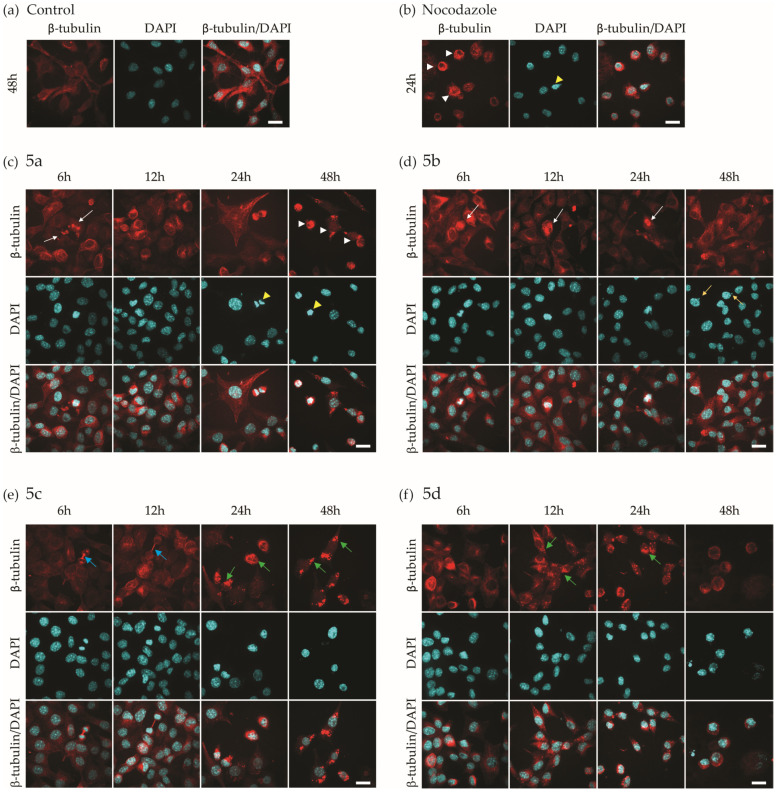
Microtubule organization (β-tubulin) and nuclear morphology (DAPI) of MDA-MB-231. (**a**) negative control—48 h incubation; (**b**) nocodazole (positive control)—24 h treatment with 0.38 µM; **5a** (**c**), **5b** (**d**), **5c** (**e**) and **5d** (**f**) after treatment with IC_50_ values (determined for 48 h treatment) of the compounds for 6, 12, 24 and 48 h. White arrowheads—abnormal cell morphology; yellow arrowheads—abnormal nuclear morphology; white arrows—mitotic spindle; yellow arrows—micronuclei; green arrows—tubulin aggregates; blue arrows—polar microtubules of the mitotic spindle. Scale bar—20 µm.

To study the effect of the compounds on microtubule organization in living cells, immunofluorescence staining was performed from the 6th to the 48th hour. While untreated control cells continued to proliferate and had normal cell morphology after 48 h (Figure 4a), exposure to nocodazole (positive control) resulted in visible cell and nuclear shape abnormalities right after 24 h (Figure 4b—white and yellow triangles).

Compound **5a** acts as a strong inhibitor of microtubule dynamics, as no cell division was observed after 6 h of treatment (Figure 4c). Moreover, the cells gradually lost their normal morphology. One of the functions of the microtubules is maintenance of the nuclear structure, which was compromised after 24 h of treatment with **5a** (Figure 4c—yellow triangles). After 48 h, the cells completely lost their normal morphology, although they were still viable (Figure 4c—white triangles). 

Compound **5b** caused no inhibition of mitotic spindle formation up to 24 h after treatment (Figure 4d—white arrows). After 48 h of treatment, cells with micronuclei were observed (Figure 4d—yellow arrows). Micronuclei are usually formed as a result of improper formation of the mitotic spindle and the uneven distribution of chromosomes, which leads to their rupture [44]. Therefore, although microtubule polymerization is not completely inhibited, it is visibly affected by compound **5b** after 48 h. We obtained similar results for this compound in an earlier study, but for a slightly different time duration [43].

In comparison, cells treated with compound **5c** underwent mitosis within the first 6 h of treatment, as shown by polar microtubules of the mitotic spindle in telophase (Figure 4e—blue arrows). These structures were still visible and appeared elongated after 12 h, suggesting a blockage in tubulin depolymerization. Moreover, cytoplasmic tubulin aggregations similar to those seen after treatment with **5d** were also present 24 h post-treatment (Figure 4e—green arrows). 

The most cytotoxic compound **5d** caused blockage of mitotic phases during the whole time frame of the immunofluorescence staining experiment. Cytoplasmic tubulin aggregates were visible 12 h after treatment (Figure 4f—green arrows), which might explain the inability of the cells to undergo mitosis. Following 48 h of incubation, the cell and nuclear morphology were severely altered.

The observed changes in mitosis occurrence, nuclear and cellular shape and tubulin structures after treatment with compounds **5a**–**d** are summarized in Table 3.

These results indicate that compound **5d** is most prominent in disrupting microtubule organization and blocking mitosis in MDA-MB-231 cells. Compound **5a** showed an abnormal mitotic spindle and irregular nuclear and cell shape and caused delayed mitotic arrest. The abnormalities observed in the microtubules and the presence of micronuclei in cells treated with **5c** and **5b** later (after 12/24 h) also led to mitotic arrest.

## 3. Materials and Methods

### 3.1. Reagents and Materials

The synthetic chemicals (o-phenylenediamine, 98%; 3,4-diaminotoluene, 97%; and the benzaldehydes, 2,3-dihydroxybenzaldehyde and 3,4,5-trimethoxybenzaldehyde) were purchased from Sigma Aldrich (St. Louis, MO, USA) and Alfa-Aesar (Ward Hill, MA, USA). 

Thin-layer chromatography (TLC) was used for monitoring the reactions performed on ALUGRAM SIL G/UV254 pre-coated aluminum sheets with silica gel 60, 0.20 mm thick (Macherey-Nagel, Düren, Nordrhein-Westfalen, Germany), eluted by toluene–methanol (3:1, *v*/*v*) and visualized by UV light (λ = 254 nm). The melting point (mp) values were determined using a Büchi B-540 instrument (Büchi Labortechnik AG, Flawil, Switzerland) and were not corrected. The compounds were characterized by FT-IR spectroscopy, and the spectra were obtained on a Bruker Tensor 27 FT spectrometer (Billerica, Massachusetts, USA) in solid state in an ATR state on a diamond accessory, with 64 scans at a 2 cm^−1^ resolution. ^1^H- and ^13^C-NMR spectra were registered on a Bruker Avance II+ 600 MHz NMR instrument (Billerica, Massachusetts, USA) in DMSO-d_6_ solvent. Chemical shifts (δ) are reported in parts per million (ppm); coupling constants (J) are given in Hz; and splitting patterns are shown as s (singlets), d (doublets), t (triplets) and m (multiplets). The mass spectra were recorded using a Thermo Scientific Q Exactive Plus Hybrid Quadrupole-Orbitrap Mass Spectrometer (Waltham, MA, USA) (HESI HRMS) in positive and negative mode. The spectra were processed using the Thermo Scientific FreeStyle program version 1.8 SP1 (Thermo Fisher Scientific Inc., Waltham, MA, USA).

### 3.2. Synthesis

The methodology for the synthesis of 1*H*-benzimidazol-2-yl-thiols **2.1**–**2.2**, 1*H*-benzimidazol-2-yl-sulfonic acids **3.1**–**3.2** as well as 1*H*-benzimidazol-2-yl-hydrazides **4.1**–**4.2** was described in previous publications [27,28]. 

The general procedure for preparation of 2-[2-benzylidenehydrazinyl]-1*H*-benzimidazole **5a**–**d** was the following: 2,3-dihydroxy- or 3,4,5-trimethoxybenzaldehyde (0.002 mol) was added to a mixture of an equimolar amount of 5(6)-methyl-1*H*-benzimidazol-2-yl-hydrazine **4.2** (0.002 mol) in absolute ethanol (5 mL); then, the solution was refluxed until the reaction was completed (appr. 3–4 h). TLC (toluene/methanol = 3:1) was used for monitoring the reaction. The precipitated product was filtered off, washed with ethanol and recrystallized twice from ethanol.

A detailed description of the synthesis of **5a** and **5b** is provided in [28].

2-(2,3-Dihydroxybenzylidene)-1-(1*H*-benzimidazol-2-yl)hydrazine (**5a**):

Yield: 70%; Mp 298.0–300.4 °C; Rf = 0.40 (toluene/methanol = 3:1, *v*/*v*); IR (ν*_max_*/cm^−1^): 3403 (νO-H), 3382 (νN-H), 3060 (νCHarom), 1628 (δNH), 1614 (νC=N), 1262, 1212 (νAr-O); 720, 710 (γC-H); ^1^H NMR (600 MHz, DMSO-*d_6_*) δ (ppm): 11.50 (bs, 1H, NH), 9.33 (s, 1H, OH), 8.30 (s, 1H, N=CH), 7.21–7.18 (m, 3H, Ar-bz; Ar), 6.95 (s, 2H, Ar-bz), 6.78–6.76 (dd, *J* = 7.75 Hz, 1.52 Hz, 1H, Ar), 6.70–6.67 (t, *J* = 15.54, 7.72 Hz, 1H, Ar); ^13^C NMR (150 MHz, DMSO*-d_6_*) δ (ppm): 145.92, 145.01, 121.50, 119.42, 118.31, 116.15. HRMS (ESI) *m*/*z*: calcd: [M+H]^+^ 269.1033; [M-H]^+^ 267.0887. Found: [M+H]^+^ 269.1031, [M-H]^+^ 267.0888.

2-(3,4,5-Trimethoxybenzylidene)-1-(1*H*-benzimidazol-2-yl)hydrazine (**5b**): 

Yield: 60%; Mp 216.1–218.5 °C; Rf = 0.50 (toluene/methanol = 3:1, *v*/*v*); IR (ν*_max_*/cm^−1^): 3380 (νN-H), 3060, 3005 (νCHarom), 2940 (ν^as^CH_3_), 2834 (ν^s^CH_3_), 1655 (δNH, νC=N), 1612 (νC=N), 1462 (δ^as^CH_3_), 1359 (δ^s^CH_3_), 1228, 1121 (νC-O-C), 728 (γC-H); ^1^H NMR (600 MHz, DMSO-*d_6_*) δ (ppm): 11.55 (bs, 2H, NH), 7.95 (s, 1H, N=CH), 7.27–7.25 (m, 2H, Ar-bz), 7.11 (s, 2H, Ar), 6.99–6.95 (m, 2H, Ar-bz), 3.86 (s, 6H, OCH_3_), 3.69 (s, 3H, OCH_3_); ^13^C NMR (150 MHz, DMSO-*d_6_*) δ (ppm): 153.76, 141.38, 138.76, 131.08, 104.35, 60.58, 56.52. HRMS (ESI) *m*/*z*: calcd: [M+H]^+^ 327.1451; [M-H]^+^ 325.1306. Found: [M+H]^+^ 327.1448; [M-H]^+^ 325.1304.

2-(2,3-Dihydroxybenzylidene)-1-(5(6)-methyl-1*H*-benzimidazol-2-yl)hydrazine (**5c**): 

Yield: 86%; Mp 285.0–287.1 °C; Rf = 0.44 (toluene/methanol = 3:1, *v*/*v*); IR (ν*_max_*/cm^−1^): 3378 νN-H), 3058 (νCHarom), 2911 (ν^as^CH_3_), 2850 (ν^s^CH_3_), 1649 (δNH), 1614 (νC=N), 1467 (δ^as^CH_3_), 1381 (δ^s^CH_3_), 1261 (νAr-O), 715 (γC-H); ^1^H NMR (600 MHz, DMSO-*d_6_*) δ (ppm): 11.36 (bs, 1H, NH), 9.34 (s, 1H, OH), 8.28 (s, 1H, N=CH), 7.17–7.16 (d, *J* = 7.47 Hz, 1H, Ar-bz), 7.06–7.04 (d, *J* = 7.82 Hz, 1H, Ar-bz), 6.99 (s, 1H, Ar-bz), 6.77–6.75 (m, 2H, Ar), 6.69–6.67 (t, *J* = 7.73, 15.52 Hz, 1H, Ar), 2.32 (s, 3H, CH_3_-bz); ^13^C NMR (150 MHz, DMSO-*d_6_*) δ (ppm): 145.90, 145.04,142.41, 121.44, 119.41, 118.55, 116.14, 21.69. HRMS (ESI) *m*/*z*: calcd: [M+H]^+^ 283.1189; [M-H]^+^ 281.1044. Found: [M+H]^+^ 283.1187; [M-H]^+^ 281.1043.

2-(3,4,5-Trimethoxybenzylidene)-1-(5(6)-methyl-1*H*-benzimidazol-2-yl)hydrazine (**5d**): 

Yield: 75%; Mp 224.6–227 °C; Rf = 0.54 (toluene/methanol = 3:1, *v*/*v*); IR (ν*_max_*/cm^−1^): 3257 (νN-H), 3012 (νCHarom), 2958 (ν^as^CH_3_), 2848 (ν^s^CH_3_), 1648 (δNH), 1612 (νC=N), 1458 (δ^as^CH_3_), 1381 (δ^s^CH_3_), 1271, 1047 (νC-O-C), 799 (γC-H); ^1^H NMR (600 MHz, DMSO-*d_6_*) δ (ppm): 11.38 (bs, 1H, NH), 7.95 (s, 1H, N=CH), 7.17–7.05 (m, 4H, Ar, Ar-bz), 6.82–6.76 (m, 1H, Ar-bz), 3.89–3.70 (d, *J* = 7.87 Hz, 9H, OCH_3_), 2.35 (s, 3H, CH_3_-bz); ^13^C NMR (150 MHz, DMSO-*d_6_*) δ (ppm): 153.60, 141.28, 138.69, 131.18, 104.29, 60.59, 56.50, 21.70. calcd: [M+H]^+^ 341.1608; [M-H]^+^ 339.1462. Found: [M+H]^+^ 341.1602; [M-H]^+^ 399.1459.

### 3.3. In Vitro Tubulin Polymerization Assay

A tubulin polymerization assay was carried out with purified porcine tubulin using a commercial assay kit (cat. number BK006P, Cytoskeleton, Denver, CO, USA). Beforehand, a fresh buffer solution (PB-GTP) containing polymerization buffer (100 µL volume of 3 mg·mL^−1^ tubulin in 80 mM piperazine-N,N′-bis(2-ethanesulfonic acid) (PIPES) pH 6.9, 0.5 mM EGTA, 2 mM MgCl, 1 mM GTP, 10% glycerol) was prepared. Then, a stock solution of pure bovine tubulin (240 µM) dissolved in the PB-GTP was diluted four-fold to obtain a 60 µM tubulin concentration before the addition of the tested compounds. Stock solutions of the test samples were prepared at a concentration of 700 µM in dimethylsulfoxide (DMSO). Paclitaxel (700 µM) and nocodazole (700 µM) in DMSO were used as positive controls. The test and control samples were adjusted to a final concentration of 70 µM by dissolution in PB-GTP. In a 96-well plate kept on ice, 60 µL of 60 µM tubulin and 10 µL of 70 µM of the test samples or positive control were applied in each well. The spontaneous polymerization of tubulin was studied by the addition of 10 µL buffer to 60 µL of 60 µM tubulin. The reaction microplate was incubated at 37 °C in the thermostated spectrophotometer chamber. The turbidity of the mixtures was monitored at 350 nm every 30 s for 90 min and compared to nocodazole and paclitaxel used as negative and positive controls. For registering the turbidity, a SpectroStar Nano (BMG Labtech, Ortenberg, Baden-Württemberg, Germany) equipped with software for kinetic measurements was used.

### 3.4. Cell Lines

To determine the cytotoxic potential of the selected compounds, the human epithelial breast cancer cell line MDA-MB-231 (American Type Culture Collection (ATCC), Manassas, VA, USA) was used. The cells were cultured in DMEM (Dulbecco’s modified essential media) (cat. No. 30-2002, ATCC, Manassas, VA, USA) with 10% fetal bovine serum (FBS) (cat. No. F7524, Sigma-Aldrich Co. LLC, St. Louis, MO, USA), 1 mM L-glutamine (cat. No. G7513, Sigma-Aldrich Co. LLC, St. Louis, MO, USA) and penicillin/streptomycin/amphotericin B (100 units/mL: 10 mg/mL: 25 μg/mL) (cat. No. A5955, Sigma-Aldrich Co. LLC, St. Louis, MO, USA) at 37 °C, 5% CO_2_ and optimal humidity.

### 3.5. Cell Viability Assay

The cell viability was measured via the (3-(4,5-dimethylthiazol-2-yl)-2,5-diphenyl tetrazolium bromide (MTT, cat. No. 475989, Calbiochem, Merck KGaA, Darmstadt, Germany) assay [45]. Briefly, the cells were trypsinized after reaching 80–90% confluence, seeded in 96-well plates with a concentration of 1 × 10^4^ cells/well and left to adhere for 24 h. After the incubation period, the cells were treated with compounds **5a**, **5b**, **5c** and **5d** at concentrations ranging from 1 to 150 µM for 48 and 72 h. Cells treated with DMSO at a concentration corresponding to the highest concentration in the samples (0.71%) were used as a solvent control. Untreated cells were used as a negative control. After the incubation period, 20 µL of MTT reagent (5 mg/mL) was added to the cells, which were incubated for 3 h at 37 °C and 5% CO_2_ and then dissolved in 5% formic acid in isopropanol. Absorbance was detected at 570 nm on a Tecan Infinite F200 PRO plate reader (Tecan GmbH, Salzburg, Austria). The cytotoxicity of the selected compounds was reflected by cell viability as a percentage of the negative control. The half-maximal inhibitory concentration (IC_50_) was determined using the GraphPad Prism 5 program (GraphPad Software, San Diego, CA, USA). The IC_50_ value of compound **5b** for 72 hours’ incubation was estimated earlier [43].

### 3.6. Immunofluorescence

Immunofluorescence of β-tubulin was performed in order to determine the in vitro effect of the selected compounds on microtubule organization in the MDA-MB-231 cell line. Briefly, cells were seeded on cover slides in 24-well plates at a concentration of 5 × 10^4^ cells/well and incubated for 24 h until complete adhesion. The cells were then treated with the pre-determined 48 h IC_50_ concentrations of compounds **5a**, **5b**, **5c** and **5d**. To monitor the effect of the compounds over time, incubation time frames of 6, 12, 24 and 48 h were used. A positive control of cells exposed to 0.38 µM nocodazole (Cat. No. CS210568, Merck Millipore, MA, USA) [23] for 24 h was also used. After the allotted treatment time, the cells were fixed with 4% paraformaldehyde; permeabilized with 0.5% Triton X-100; blocked with 1% bovine serum albumin (BSA) in phosphate buffer saline, pH 7.4, with 0.1% Tween 20; and labeled with rabbit monoclonal antibody against β-tubulin (1:500, Cat. No. 32-2600, Invitrogen, Waltham, MA, USA) for 1 h at room temperature. A donkey anti-rabbit secondary antibody conjugated to TRITC (1:1000, Cat. No. sc-2781, Santa Cruz Biotechnology, Dallas, TX, USA) was attached to the primary antibody. Finally, cell nuclei were stained for 5 min at room temperature in the dark with 1 µg/mL 4′,6-diamidino-2-phenylindole (DAPI, D9542, Sigma-Aldrich Co. LLC, St. Louis, MO, USA). Fluorescence microscopy was performed on a spinning disk confocal microscope (Andor Revolution XD) at 63× magnification. The obtained data were analyzed with Fiji [46].

### 3.7. Molecular Docking and DFT Calculation

The Molecular Operating Environment (MOE) 2022 software package [38] was used for the molecular docking. The ligands were docked in the structure of αβ-tubulin with the stathmin-like domain of the RB3 protein in complex with colchicine and vinblastine, obtained by XRD with an overall resolution of 4.10 Å resolution, PDB ID: 1Z2B [31]. The structure was protonated (pH 7.0, 300 K, Salt 0.1 M/L) using the 3D protonation algorithm implemented in the MOE package. Different conformations of the studied compounds were included in the docking study accounting for amino and imino tautomeric forms, E and Z configuration of the azomethyne double bond, s-cis and s-trans arrangement of the substituents around the N-N bond, formation of intramolecular hydrogen bonds, etc. The Triangle Matcher algorithm was used for the initial placement of the ligand conformations, and the resulting poses were scored by the London dG [30] function. The best 50 poses for every ligand were further optimized with the Induced Fit methodology, using the MMFF94x force field/Born solvation model and optimization cutoff of 6 Å from the ligand. The GBVI/WSA dG [30] was used as a rescoring function, and the best 30 poses were collected for further analysis.

The molecular geometry of hydrazones **5c** and **5d** was examined using the Gaussian 09 [47] program package with density functional theory (DFT). For this purpose, the hybrid functional B3LYP [48] and 6-311++G** basis set [49,50] was implemented. The solvent effects in water and DMSO were included through the Polarizable Continuum Model (PCM) [51]. ^1^H- and ^13^C-NMR chemical shifts in DMSO solvent were estimated by the GIAO method [52] at the B3LYP/6-311++G** level of theory. The chemical shifts for the tetramethylsilane reference compound (TMS) were calculated at the same level of theory.

## 4. Conclusions

In this work, we assessed the antiproliferative potential of four benzimidazole derivatives against the triple-negative breast cancer cell line MDA-MB-231. While all tested compounds (**5a**–**d**) exhibited weaker cell growth inhibition than the positive control nocodazole, compounds **5c** and **5d** demonstrated notable antiproliferative effects after extended treatment. Particularly, compound **5d**, distinguished by a methyl group at the 5(6)-position of the benzimidazole ring and a colchicine-like fragment in the hydrazone chain, significantly hindered tubulin polymerization and induced early mitotic arrest. This was corroborated by molecular docking studies, suggesting that the interaction at the colchicine binding site of the ligands primarily involves van der Waals forces, with **5d** also showing lipophilic interactions of the trimethoxyphenyl ring, with Lys254 from β-tubulin contributing to its enhanced activity. After 48 h of treatment, pronounced disruption of cell morphology and nuclear integrity was observed, leading to the presence of only cell remnants. Notably, **5d** significantly slowed down the initial rate of tubulin polymerization and was comparable to nocodazole. 

## Figures and Tables

**Table 1:** In vitro effect on tubulin polymerization of 1*H*-benzimidazol-2-yl hydrazones **5a**–**d**, paclitaxel and nocodazole.

Compound	Tubulin Polymerization ^1^	
	Lag Time, s	Initial Rate ^2^
Tubulin (spontaneous polymerization)	no lag phase	75.3
Unsubstituted benzimidazole ring		
**5a** ^3^	1461	13.4
**5b** ^3^	988	20.6
5(6)-Methyl benzimidazole ring		
**5c**	no lag phase	27.0
**5d**	600	12.9
Reference compounds		
Paclitaxel ^3^	151	167
Nocodazole ^3^	935	52

^1^ at final conc. of all compounds and standards 10 µM; ^2^ signal response a.u.s^−1^.10^−6^; ^3^ data from [29].

**Table 3:** Cellular characteristics after treatment with compounds over 48 hours.

Compound	Characteristics			
	Mitosis	Morphology		Tubulin
		Nucleus	Cell	
Unsubstituted benzimidazole ring				
**5a**	Until 6 h	Abnormal shape	Abnormal shape	Abnormal mitotic spindle
**5b**	Until 24 h	Micronuclei	Not affected	Not directly observed
5(6)-Methyl benzimidazole ring				
**5c**	Until 12 h	Not affected	Not affected	Elongated polar microtubules, cytoplasmic granules
**5d**	Arrested in mitosis	Abnormal shape	Abnormal shape	Cytoplasmic granules
Reference compound				
Nocodazole	Arrested in mitosis ^1^	Abnormal shape	Abnormal shape	Abnormal mitotic spindle

^1^ Ref. [5].

## Data Availability

Data are available within the article.

## Supplementary Materials

The following supporting information can be downloaded at https://www.mdpi.com/article/10.3390/molecules29102400/s1: Figures S1–S6. IR and NMR spectra of compounds **5c** and **5d**; Figures S7–S10. HRMS spectra of compounds **5a**–**d**; Tables S1 and S2. Optimized molecular structure and relative Gibbs energies of hydrazone **5c** in imino and amino tautomeric form. Table S3. Summarized total Gibbs energies, relative Gibbs energies and Boltzmann population for the tautomeric and isomeric forms of compound **5c**. Tables S4 and S5. Optimized molecular structure and relative Gibbs energies of hydrazone **5d** in imino and amino tautomeric form. Table S6. Summarized total Gibbs energies, relative Gibbs energies and Boltzmann population for all tautomeric and isomeric forms of compound **5d**. Tables S7 and S8. Chemical shifts for the tautomeric and isomeric forms of compounds **5c** and **5d** predicted by GIAO method in DMSO solvent and experimental data for **5c** and **5d**. Figure S11. Tubulin (final 54 µM) in presence of PB-GTP buffer, 10 µM paclitaxel, 10 µM nocodazole and 10 µM of compounds **5a**–**d**.
