# Supplementary material for: Antiproliferative and Pro-Apoptotic Activity and Tubulin Dynamics Modulation of 1H-Benzimidazol-2-yl Hydrazones in Human Breast Cancer Cell Line MDA-MB-231
Source: Molecules. 2024 May 20;29(10):2400. doi: 10.3390/molecules29102400 (PMC11123699; doi:10.3390/molecules29102400)
Supplement: Supplementary file 1 [file molecules-29-02400-s001.zip › molecules-2943492-supplementary.pdf]

Antiproliferative, Pro-apoptotic Activity And Tubulin Dynamics Modulation of 1*H*-benzimidazol-2-yl Hydrazones in Human Breast Cancer Cell Line MDA-MB-231

Denitsa Yancheva [1,*], Maria Argirova [1], Irina Georgieva [2], Vanya Milanova [2], Maya Guncheva [1], Miroslav Rangelov [1], Nadezhda Todorova [3], Rumiana Tzoneva [2,*]

[1] Institute of Organic Chemistry with Centre of Phytochemistry, Bulgarian Academy of Sciences, Acad. G. Bonchev St., Build. 9, 1113 Sofia, Bulgaria; denitsa.pantaleeva@orgchm.bas.bg (D. Y.); maria.argirova@orgchm.bas.bg (M. A.); maya.guncheva@orgchm.bas.bg (M. G.); miroslav.rangelov@orgchm.bas.bg (M. R.);
[2] Institute of Biophysics and Biomedical Engineering, Bulgarian Academy of Sciences, Acad. G. Bonchev St., Build. 21, 1113 Sofia, Bulgaria; igeorgieva@biomed.bas.bg (I. G.); vanyamilanova99@gmail.com (V. M);
[3] Institute of Biodiversity and Ecosystem Research, Bulgarian Academy of Sciences, 2 Gagarin Str., 1113 Sofia, Bulgaria; nadeshda@abv.bg (N. T.)
* Correspondence: tzoneva@bio21.bas.bg (R. Tz.); denitsa.pantaleeva@orgchm.bas.bg (D. Y.)

Contents:

Figs. S1-S6. IR and NMR spectra of compounds **5c** and **5d** p. 3-6

Figs. S7-S10. HRMS spectra of compounds **5a-d** p. 7-10

Table S1-S2. Optimized molecular structure and relative Gibbs energies of hydrazone **5c** in imino and amino tautomeric form p. 11-13

Table S3. Summarized total Gibbs energies, relative Gibbs energies and Boltzmann population for the tautomeric and isomeric forms of compound **5c** p. 14

Table S4-S5. Optimized molecular structure and relative Gibbs energies of p. 15-17

hydrazone **5d** in imino and amino tautomeric form

Table S6. Summarized total Gibbs energies, relative Gibbs energies and Boltzmann population for all tautomeric and isomeric forms of compound **5d** p. 18

Table S7-S8. Chemical shifts for the tautomeric and isomeric forms of compounds **5c** and **5d** predicted by GIAO method in DMSO solvent and experimental data for **5c** and **5d**. p. 19-23

Fig. S11. Tubulin (final 54 μM) in presence of PB-GTP buffer, 10 μM paclitaxel, 10 μM nocodazole and 10 μM of compounds **5a-d**. p. 24

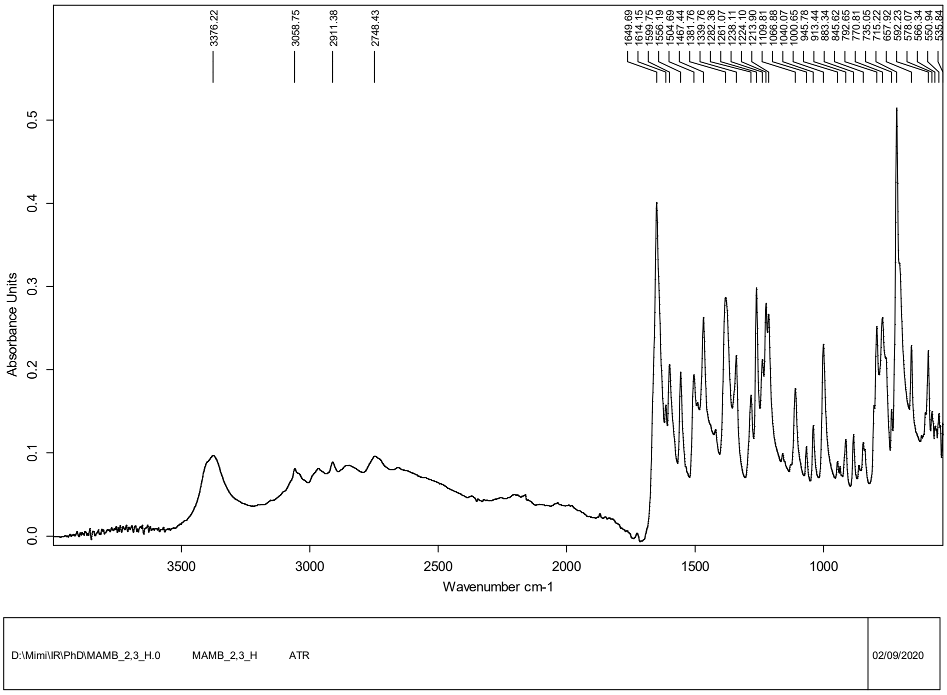

Fig. S1a. ATR-IR spectrum of compound **5c**

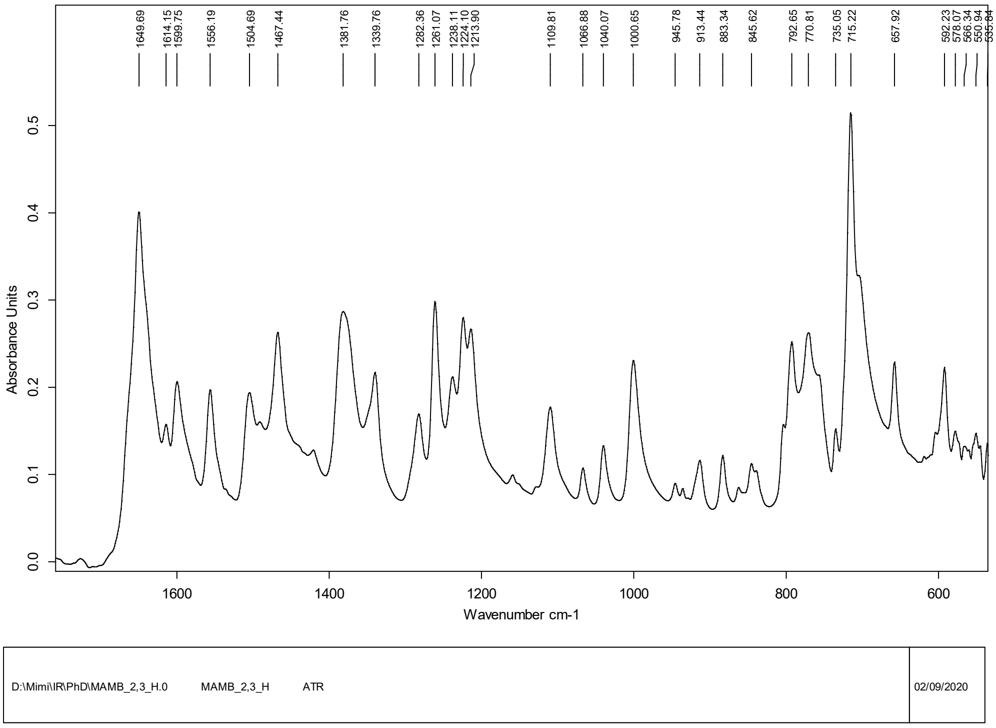

Fig. S1b. ATR-IR spectrum of compound **5c**

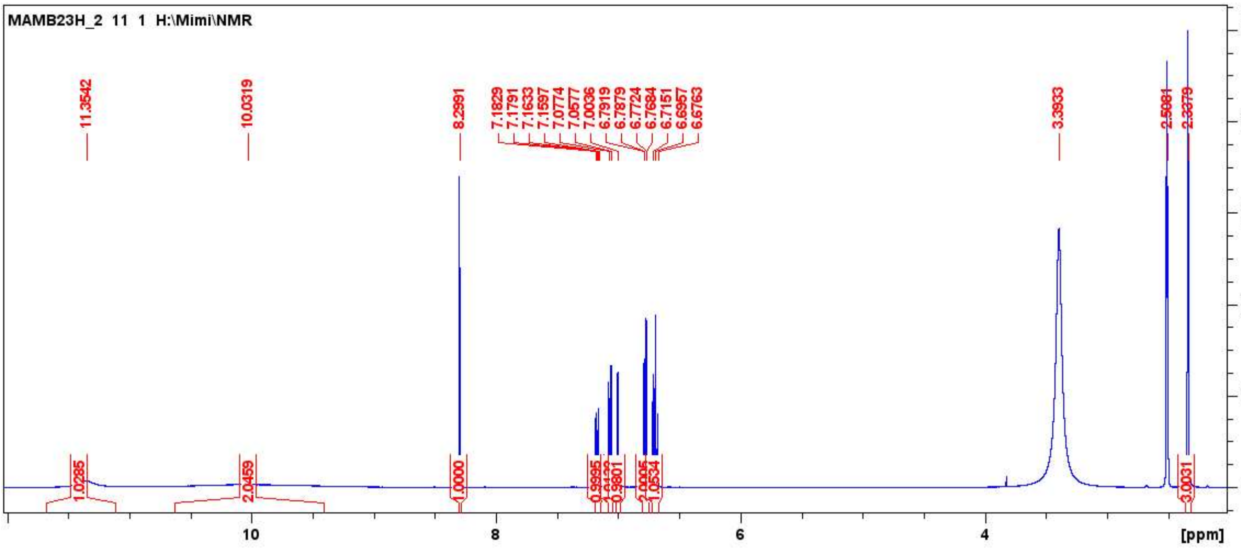

Fig. S2. ^{1}H NMR spectrum of compound **5c** in DMSO-*d*6

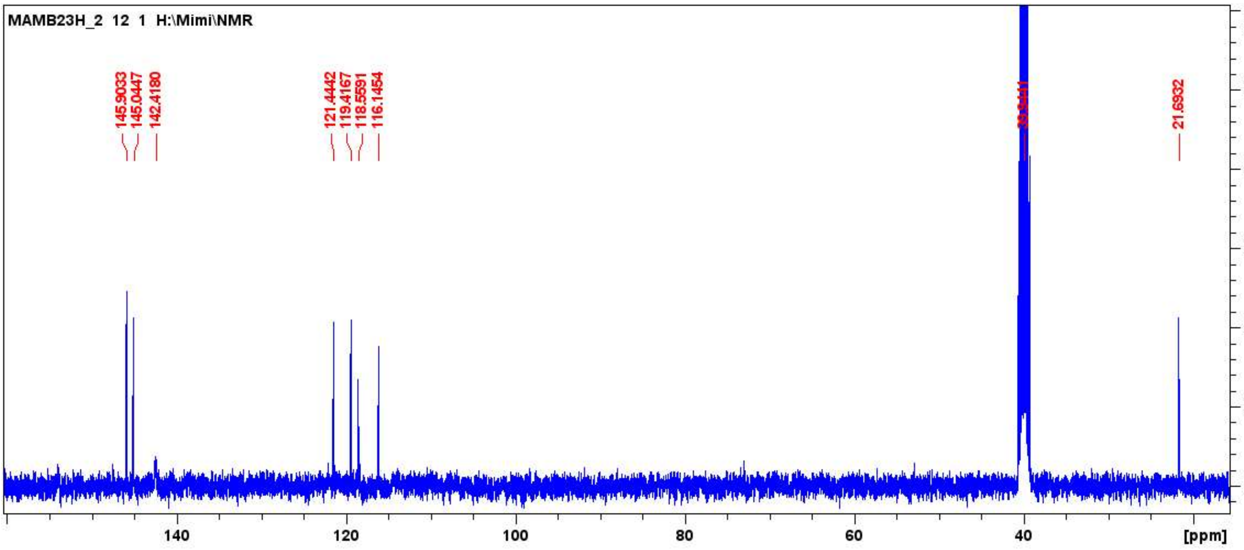

Fig. S3. ^{13}C NMR spectrum of compound **5c** in DMSO-*d*6

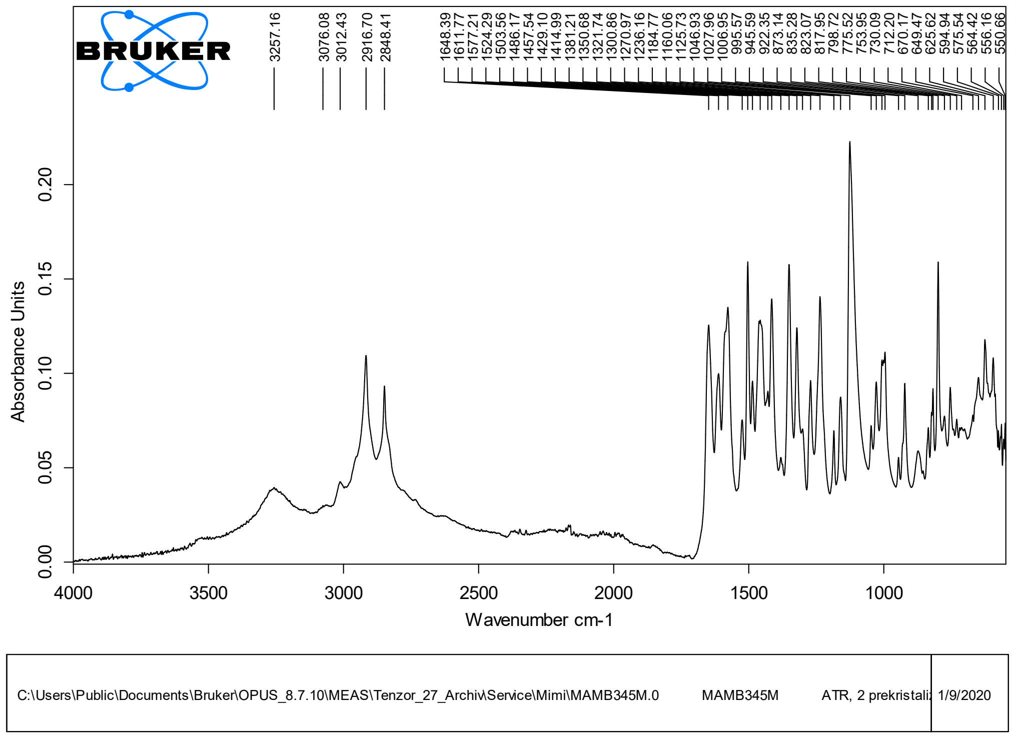

Fig. S4a. ATR-IR spectrum of compound **5d**

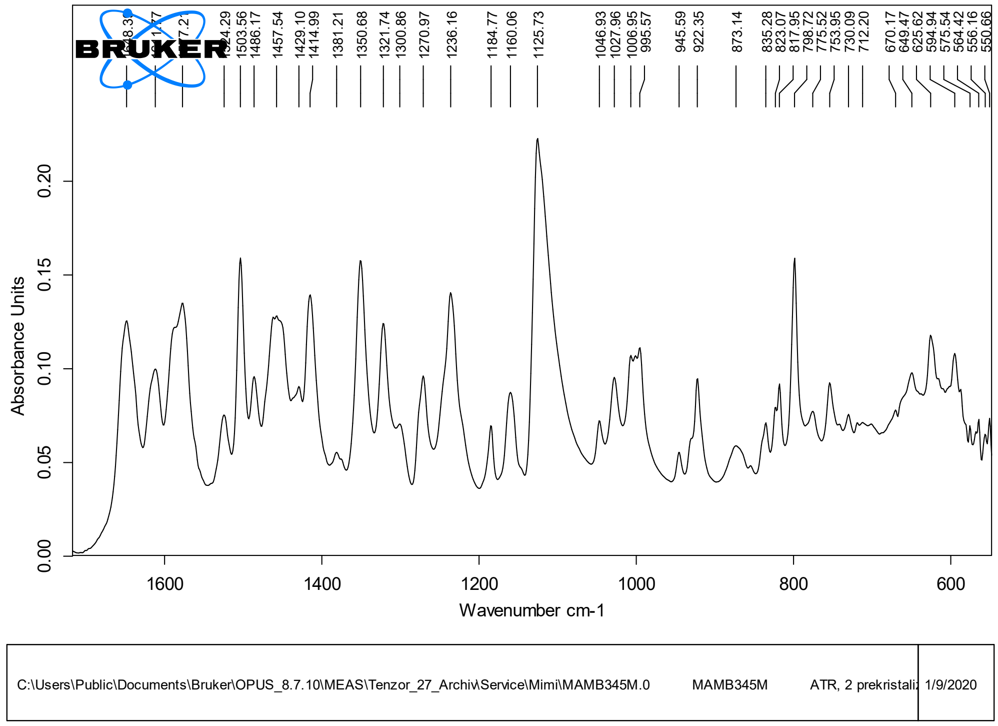

Fig. S4b. IR spectrum of compound **5d**

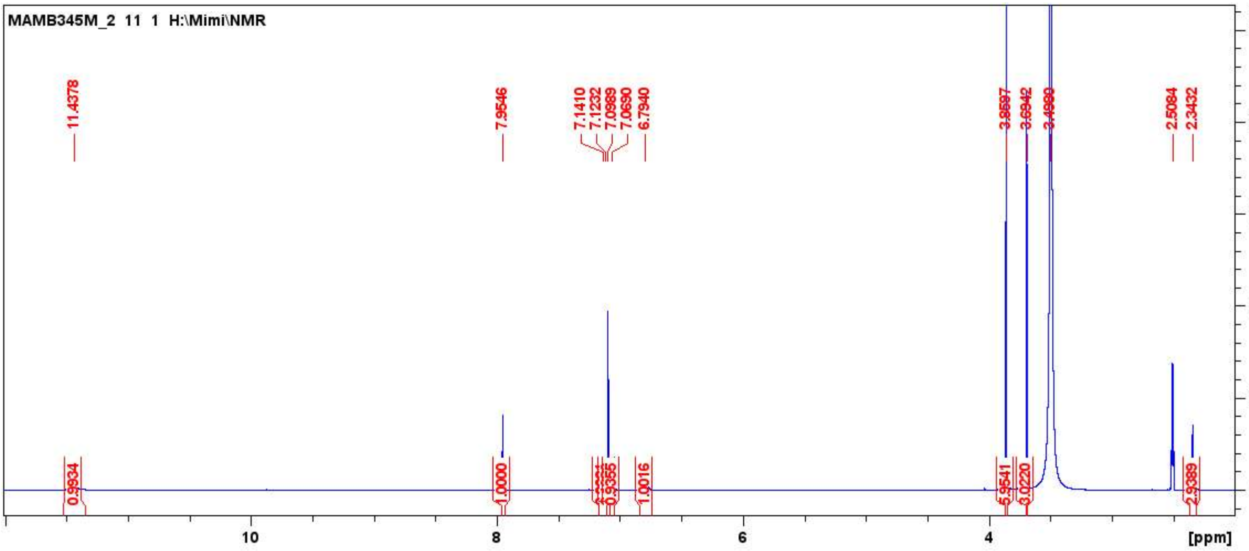

Fig. S5. ^{1}H NMR spectrum of compound **5d** in DMSO-d_6

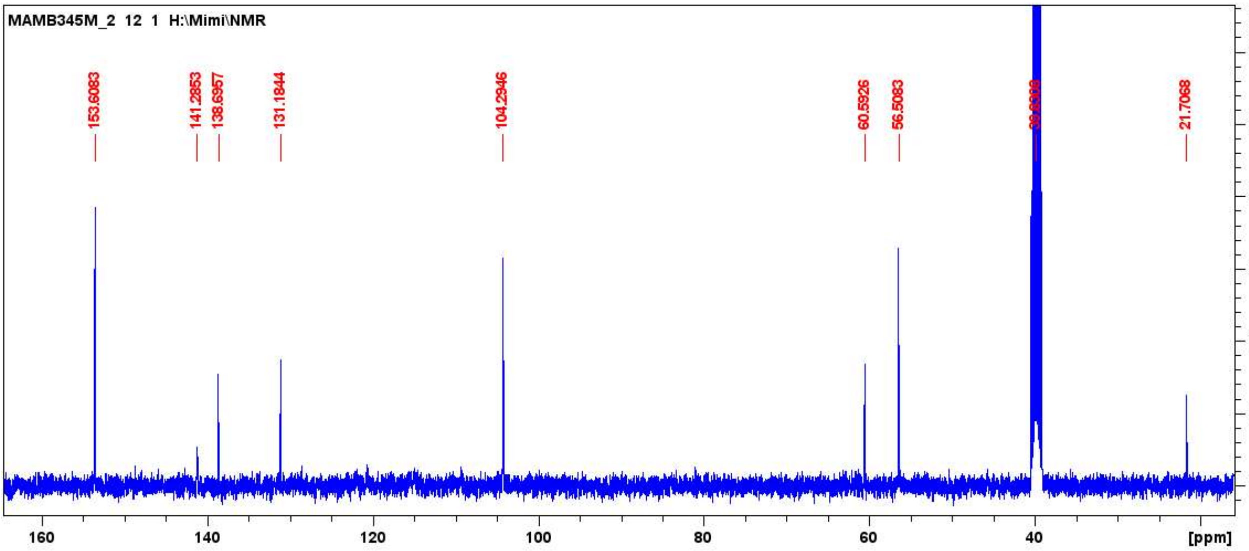

Fig. S6. ^{13}C NMR spectrum of compound **5d** in DMSO-d_6

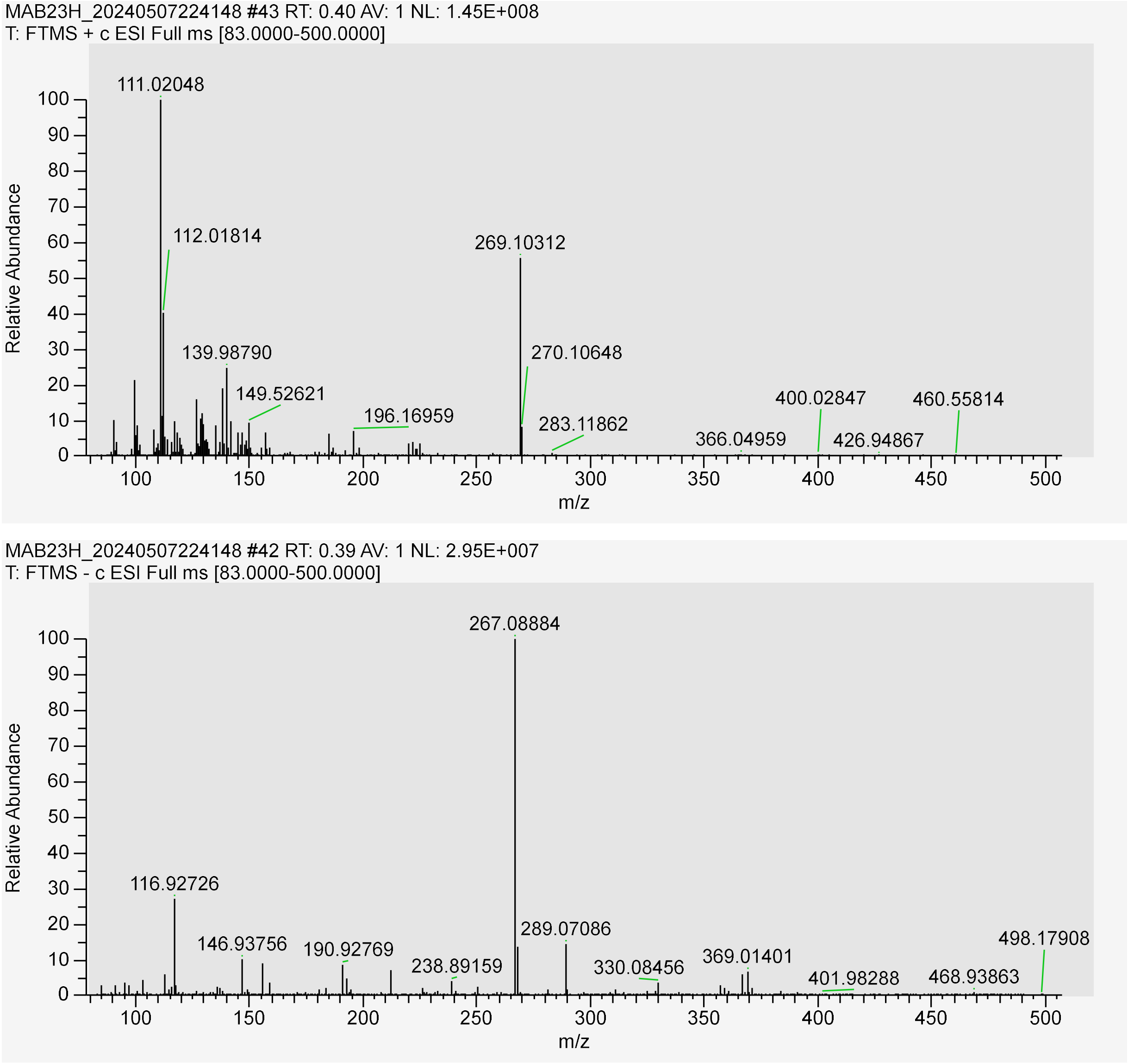

Figure S7. HRMS spectra of **5a**

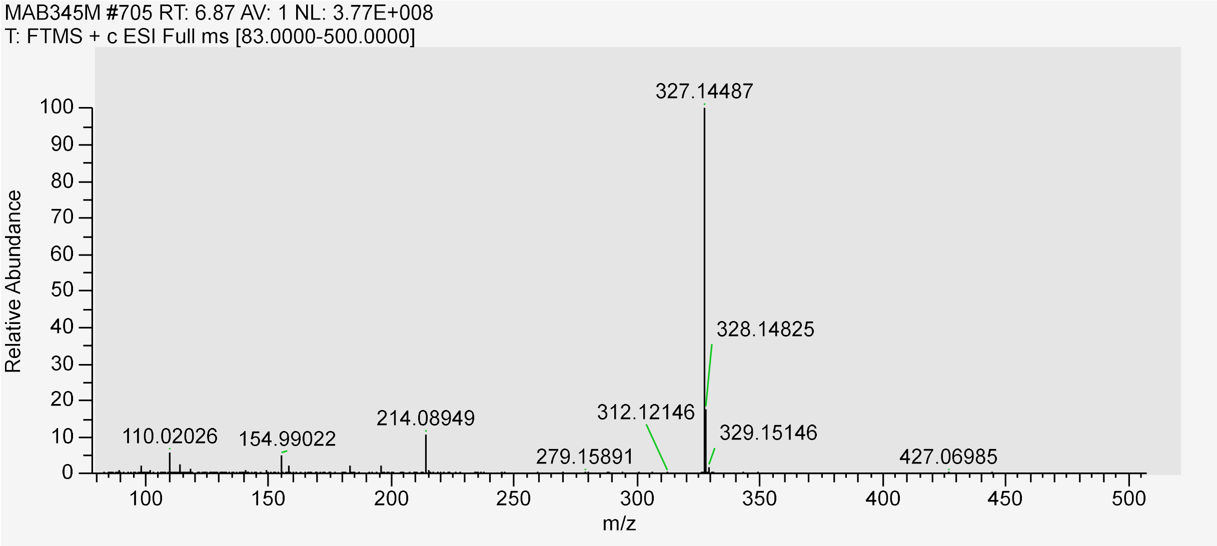

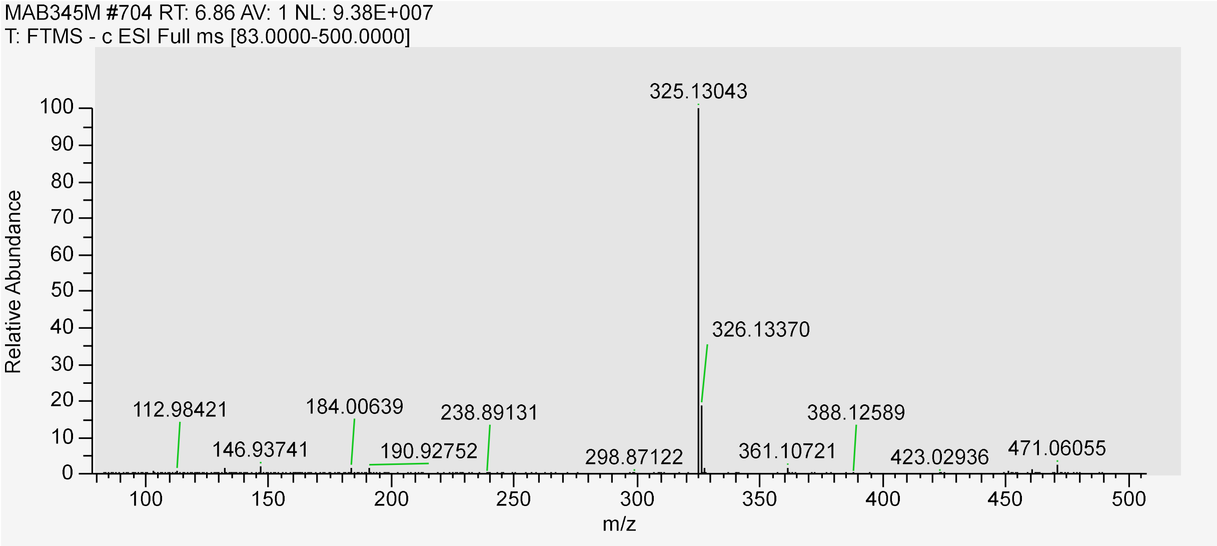

Figure S8. HRMS spectra of **5b**

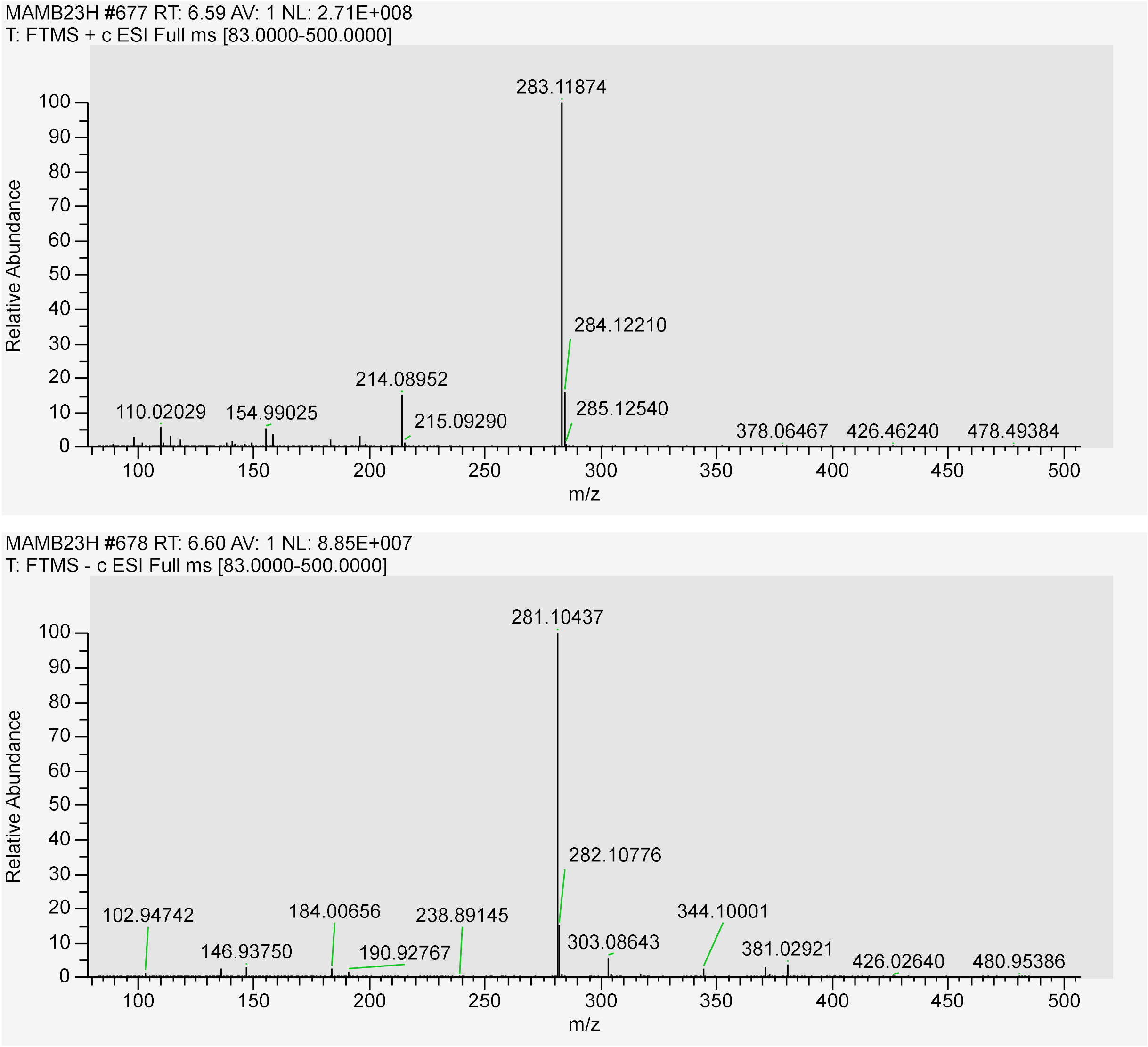

Figure S9. HRMS spectra of **5c**

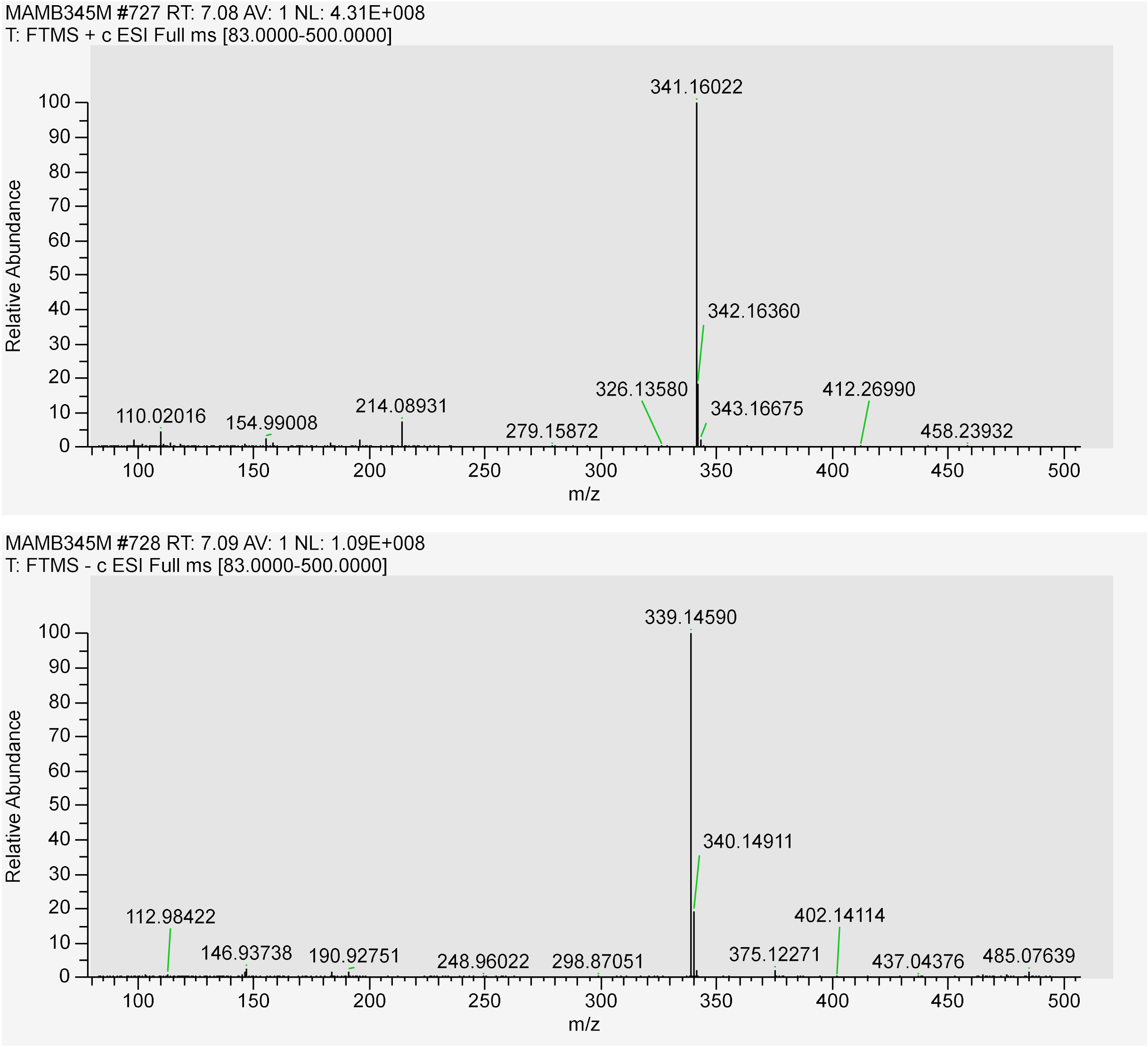

Figure S10. HRMS spectra of **5d**

Table S1. Optimized molecular structure, total Gibbs energy (in a.u.) and relative Gibbs energies (in kJ mol^{-1}) of the possible isomers of compound **5c** in imino tautomeric form, obtained at B3LYP/6-311++G(d,p) level of theory in gas phase and water.

Medium	Imino tautomeric form	ΔG
	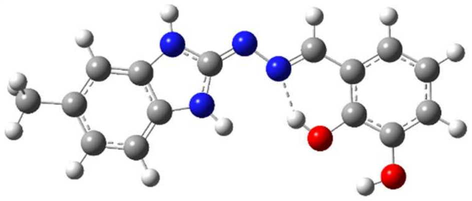	
	E, s-trans	
**gas phase**	G_{tot}: -949.516836	-
**water**	G_{tot}: -949.533431	-
	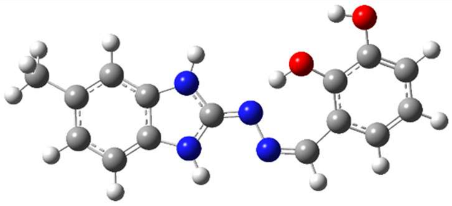	
	Z, s-trans	
**gas phase**	G_{tot}: -949.514769	5.42
**water**	G_{tot}: -949.530744	7.05

Table S2. Optimized molecular structure, total Gibbs energies (in a.u.) and relative Gibbs energies (in kJ.mol^{-1}) of the possible isomers of compound **5c** in amino tautomeric form, obtained at B3LYP/6-311++G(d,p) level of theory in gas phase and water.

**Medium**		**6-methyl isomer**	**5-methyl isomer**
		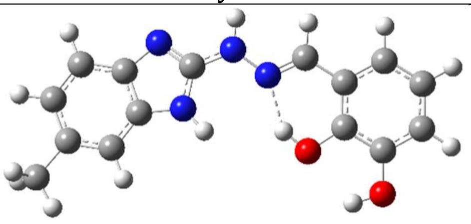	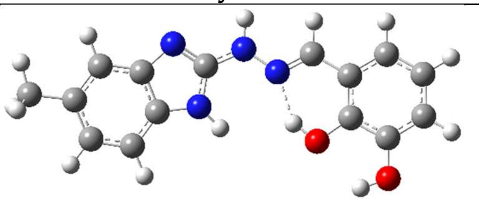
		E, s-trans	*E, s-trans*
**gas phase**	**G$_{tot}$**	-949.513467	-949.512992
	**ΔG**	-	1.24
**water**	**G$_{tot}$**	-949.531327 a.u.	-949.531095
	**ΔG**	-	0.60
		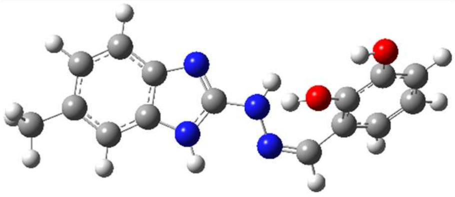	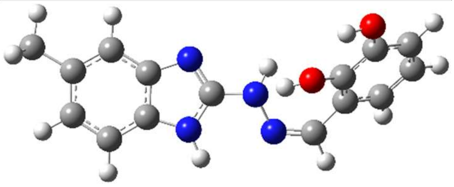
		Z, s-trans	*Z, s-trans*
**gas phase**	**G$_{tot}$**	-949.501294	-949.501873
	**ΔG**	1.52	-
**water**	**G$_{tot}$**	-949.516863	-949.51753
	**ΔG**	1.75	-
		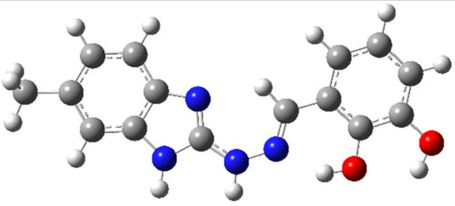	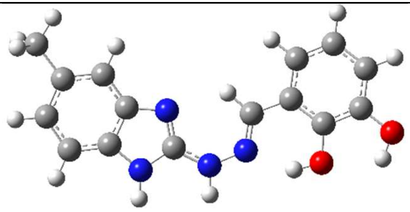
		E, s-cis	*E, s-cis*
**gas phase**	**G$_{tot}$**	-949.507653	-949.509719
	**ΔG**	5.42	-
**water**	**G$_{tot}$**	-949.525408	-949.525125
	**ΔG**	-	0.74

		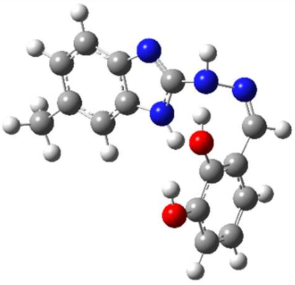	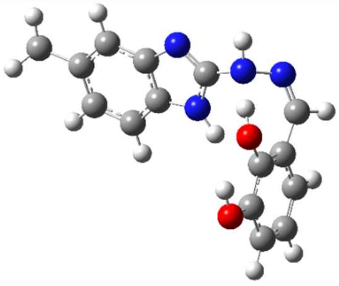
		Z, s-cis	*Z, s-cis*
**gas phase**	**G$_{tot}$**	-949.493624	-949.493092
	**ΔG**	-	1.39
**water**	**G$_{tot}$**	-949.509446	-949.509564
	**ΔG**	0.30	-

Table S3. Summarized total Gibbs energies (in a.u.), relative Gibbs energies (in a.u. and kJ.mol^{-1}) and Boltzmann population (in %) for the tautomeric and isomeric forms of compound **5c**, obtained at B3LYP/6-311++G(d,p) level of theory in different media.

**Tautomer/isomer**	G_{tot}	**ΔG (a.u.)**	**ΔG (kJ.mol^{-1})**	**Boltzmann population**
	Gas			
imino (E,s-trans)	-949.516836	0	0	86.42
imino (Z,s-trans)	-949.514769	0.002067	5.43	9.67
**6-isomer**				
amino (E,s-trans)	-949.513467	0.003369	8.85	2.43
amino (Z,s-trans)	-949.501294	0.015542	40.81	
amino (E,s-cis)	-949.507653	0.009183	24.11	
amino (Z,s-cis)	-949.493624	0.019843	52.10	
**5-isomer**				
amino (E,s-trans)	-949.512992	0.003844	10.09	1.48
amino (Z,s-trans)	-949.501873	0.014963	39.29	
amino (E,s-cis)	-949.509719	0.007117	18.69	
amino (Z,s-cis)	-949.493092	0.023744	62.34	
	Water			
imino (E,s-trans)	-949.533431	0	0	79.97
imino (Z,s-trans)	-949.530744	0.002687	7.05	4.65
**6-isomer**				
amino (E,s-trans)	-949.531327	0.002104	5.52	8.63
amino (Z,s-trans)	-949.516863	0.016568	43.50	
amino (E,s-cis)	-949.525408	0.008023	21.06	
amino (Z,s-cis)	-949.509446	0.023985	62.97	
**5-isomer**				
amino (E,s-trans)	-949.531095	0.002336	6.13	6.75
amino (Z,s-trans)	-949.51753	0.015901	41.75	
amino (E,s-cis)	-949.525125	0.008306	21.81	
amino (Z,s-cis)	-949.509564	0.023867	62.66	
	DMSO			
imino (E,s-trans)	-949.532308	0	0	53.25
**6-isomer**				
amino (E,s-trans)	-949.531449	0.000859	2.26	21.40
**5-isomer**				
amino (E,s-trans)	-949.531607	0.000701	1.84	25.35

Table S4. Optimized molecular structure, total Gibbs energy (in a.u.) and relative Gibbs energies (in kJ mol^{-1}) of the possible isomers of compound **5d** in imino tautomeric form, obtained at B3LYP/6-311++G(d,p) level of theory in gas phase and water.

Medium	Imino tautomeric form	ΔG
	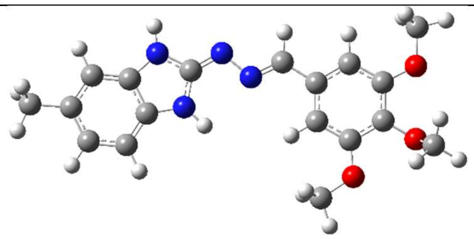	
	E, s-trans	
**gas phase**	G_{tot}: -1142,585534	-
**water**	G_{tot}: -1142,605514	-
	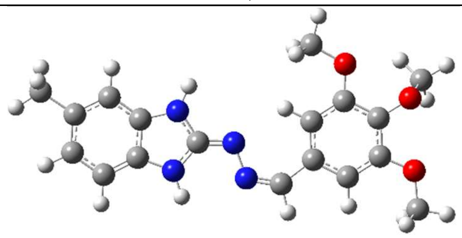	
	Z, s-trans	
**gas phase**	G_{tot}: -1142,582568	7.78
**water**	G_{tot}: -1142,604003	15.43

Table S5. Optimized molecular structure, total Gibbs energies (in a.u.) and relative Gibbs energies (in kJ.mol-1) of the possible isomers of compound **5d** in amino tautomeric form, obtained at B3LYP/6-311++G(d,p) level of theory in gas phase and water.

**Medium**		**6-methyl isomer**	**5-methyl isomer**
		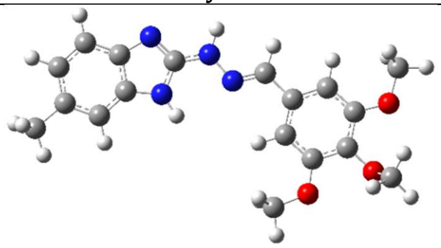	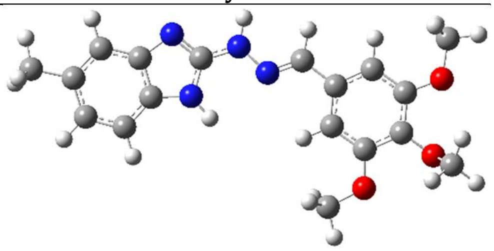
		E, s-trans	*E, s-trans*
**gas phase**	**G_{tot}**	-1142.589796	-1142.589791
	**ΔG**	-	0.01
**water**	**G_{tot}**	-1142.610250	-1142.611019
	**ΔG**	-	2.01
		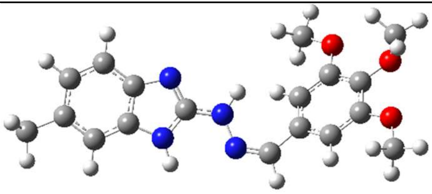	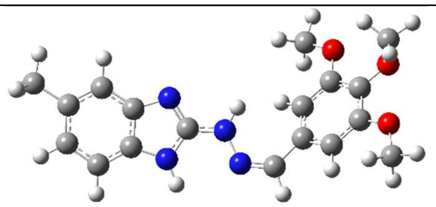
		Z, s-trans	*Z, s-trans*
**gas phase**	**G_{tot}**	-1142.583297	-1142.582924.
	**ΔG**	-	0.97
**water**	**G_{tot}**	-1142.602714	-1142.602627
	**ΔG**	-	0.22
		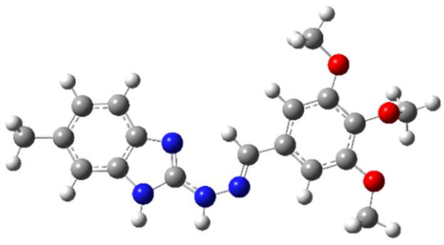	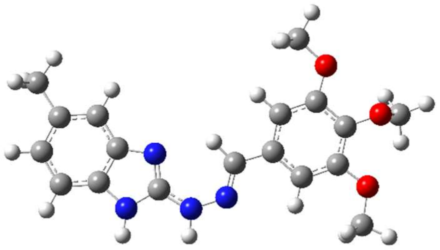
		E, s-cis	*E, s-cis*
**gas phase**	**G_{tot}**	-1142.580100	-1142.580055
	**ΔG**	-	0.11
**water**	**G_{tot}**	-1142.601929	-1142.601816
	**ΔG**	-	0.29

		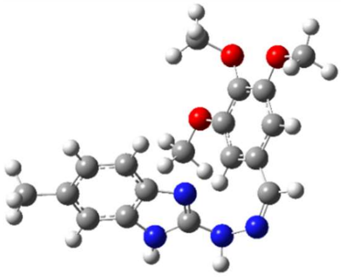	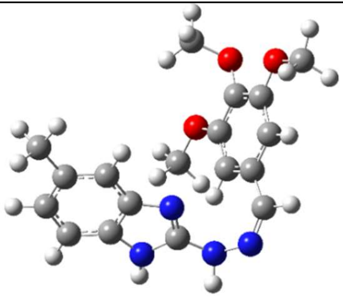
		Z, s-cis	*Z, s-cis*
**gas phase**	$\mathbf{G_{tot}}$	-1142.564804	-1142.565265
	**ΔG**	1.21	-
**water**	$\mathbf{G_{tot}}$	-1142.587979	-1142.589262
	**ΔG**	3.36	-

Table S6. Summarized total Gibbs energies (in a.u.), relative Gibbs energies (in a.u. and kJ.mol-1) and Boltzmann population (in %) for all tautomeric and isomeric forms of compound **5d**, obtained at B3LYP/6-311++G(d,p) level of theory in gas phase and water.

**Tautomer/isomer**	G_{tot}	**ΔG (a.u.)**	**ΔG (kJ.mol^{-1})**	**Boltzmann population**
		Gas		
imino (E,s-trans)	-1142.585534	0.004262	11.19	
imino (Z,s-trans)	-1142.582568	0.007228	18.98	
**6-isomer**				
amino (E,s-trans)	-1142.589796	0	0	51.10
amino (Z,s-trans)	-1142.583297	0.006499	17.06	
amino (E,s-cis)	-1142.580100	0.009696	25.46	
amino (Z,s-cis)	-1142.564804	0.024992	65.62	
**5-isomer**				
amino (E,s-trans)	-1142.589791	5.10^{-6}	0.01	49.90
amino (Z,s-trans)	-1142.582924	0.006872	18.04	
amino (E,s-cis)	-1142.580055	0.009741	25.57	
amino (Z,s-cis)	-1142.565265	0.024531	64.41	
		Water		
imino (E,s-trans)	-1142.605514	0.005505	14.45	
imino (Z,s-trans)	-1142.604003	0.007016	18.42	
**6-isomer**				
amino (E,s-trans)	-1142.610250	0.000769	2.02	30.69
amino (Z,s-trans)	-1142.602714	0.008305	21.80	
amino (E,s-cis)	-1142.601929	0.00909	23.87	
amino (Z,s-cis)	-1142.587979	0.02304	60.49	
**5-isomer**				
amino (E,s-trans)	-1142.611019	0	0	69.31
amino (Z,s-trans)	-1142.602627	0.008392	22.03	
amino (E,s-cis)	-1142.601816	0.009203	24.16	
amino (Z,s-cis)	-1142.589262	0.021757	57.12	
		DMSO		
imino (E,s-trans)	-1142.602682	0.007986	20.97	
**6-isomer**				
amino (E,s-trans)	-1142.610668	0	0	59.95
**5-isomer**				
amino (E,s-trans)	-1142.610288	0.00038	1.00	40.05

Table S7. Chemical shifts (in ppm) for the imino form, 5-methyl isomer sand 6-methyl isomer of the amino form of compound **5c** predicted by GIAO method using B3LYP functional and 6-311++G** basis set calculations in DMSO solvent and experimental data for **5c**.

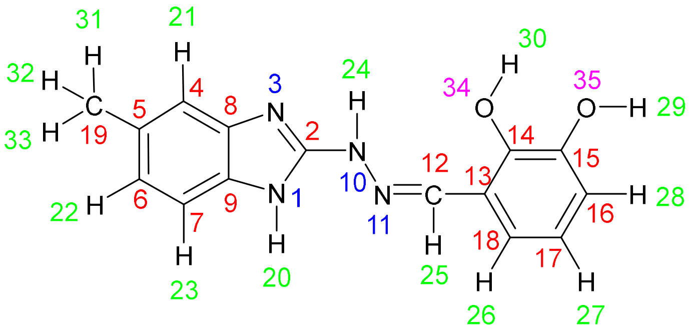

C Atom №	$\delta_{exp.}$ in DMSO-d_6	B3LYP/6-311++G** DMSO					
		Imino form		5-methyl isomer in amino form		6-methyl isomer in amino form	
		chem. shift	rel. to TMS	chem. shift	rel. to TMS	chem. shift	rel. to TMS
5	121.44	55.7767	128.8	43.6309	140.9	54.6946	129.9
4	118.55	70.5148	114.0	61.477	123.1	61.8959	122.7
6	142.41	42.6670	141.9	56.1349	128.4	44.8265	139.7
8	145.04	48.2187	136.3	33.4201	151.1	35.7915	148.8
7	116.14	69.0386	115.5	69.5873	114.9	69.0442	115.5
9	142.41	46.1206	138.4	46.1212	138.4	44.0197	140.5
2	145.90	25.3236	159.2	28.2886	156.2	28.6183	155.9
12	145.04	25.8879	158.7	34.7429	149.8	34.65	149.9
13	119.41	57.4188	127.1	59.3038	125.3	58.9294	125.6
18	121.44	57.0677	127.5	57.4073	127.1	56.7223	127.8
14	145.90	31.2122	153.4	32.7338	151.8	32.0896	152.5
17	121.44	58.167	126.4	56.7322	127.8	57.089	127.5

15	145.90	31.3152	153.2	30.4474	154.1	30.824	153.7
16	118.55	63.8011	120.8	62.0727	122.5	62.2033	122.3
19	31.69	160.9968	23.6	160.8786	23.7	160.8177	23.7
H Atom №							
21	6.99	24.7542	7.2	24.4974	7.4	24.4566	7.5
23	7.17-7.16	24.7444	7.2	24.4042	7.5	24.5319	7.4
20	11.36	24.1828	7.8	23.694	8.3	23.6377	8.3
25	8.28	24.9762	6.9	23.9583	8.0	23.8575	8.1
24	-	23.32	8.6	23.9835	7.9	23.9284	8.0
22	7.06-7.04	24.8098	7.1	24.8379	7.1	24.571	7.4
27	6.69-6.67	24.9239	7.0	21.6753	10.3	24.8774	7.1
30	9.34	20.0768	11.9	24.8807	7.1	21.6214	10.3
26	6.77-6.75	24.9005	7.0	24.6416	7.3	24.9113	7.1
28	6.77-6.75	24.8502	7.1	26.3282	5.6	24.7245	7.2
29	9.34	26.3549	5.6	29.3535	2.6	26.2931	5.7
32	2.32	29.3897	2.6	29.7879	2.1	29.2003	2.7
31	2.32	29.396	2.6	29.3012	2.6	29.2892	2.7
33	2.32	29.8073	2.1	24.6493	7.3	29.8911	2.1

Table S8. Chemical shifts (in ppm) for the 5-methyl isomer sand 6-methyl isomer of the amino form of compound **5d** predicted by GIAO method using B3LYP functional and 6-311++G** basis set calculations in DMSO solvent and experimental data for **5d**.

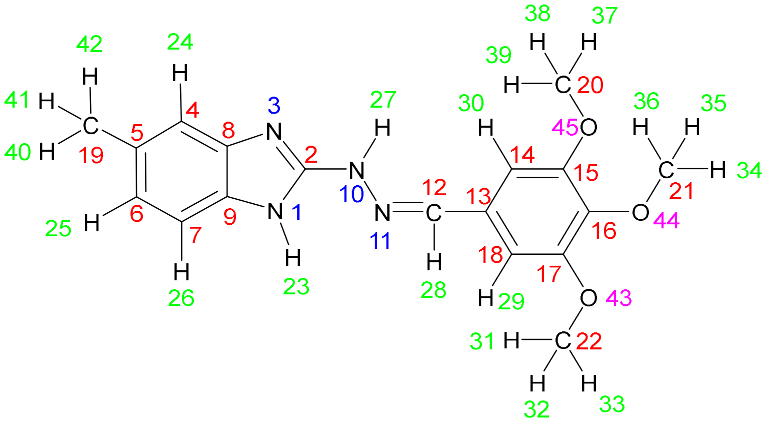

C Atom №	δExp. in DMSO-d_6	6-methyl isomer in amino form		5-methyl isomer in amino form	
		chem. shift	rel. to TMS	chem. shift	rel. to TMS
5	131.18	55.0135	129.5	43.9386	140.6
4	131.18	61.8759	122.7	61.7245	122.8
6	138.69	45.0612	139.5	57.0824	127.5
8	153.60	35.3683	149.2	32.6431	151.9
7	104.29	69.3288	115.2	69.9332	114.6
9	138.69	44.0731	140.5	46.0336	138.5
2	153.60	26.9442	157.6	26.7588	157.8
12	141.28	37.8856	146.7	37.6092	146.9
13	138.69	45.8021	138.8	46.2601	138.3
18	104.29	82.4458	102.1	82.2531	102.3
14	104.29	74.7445	109.8	73.3204	111.2
17	153.60	20.4700	164.1	20.3526	164.2

15	153.60	21.3767	163.2	20.9407	163.6
16	141.28	37.7587	146.8	36.8681	147.7
20	56.50	126.9329	57.6	126.3548	58.2
22	56.50	126.2986	58.3	125.771	58.8
21	60.59	121.2851	63.3	121.3134	63.2
19	21.70	160.8036	23.8	160.6998	23.9
H Atom №					
24	7.17-7.05	24.5066	7.4	24.5080	7.4
26	7.17-7.05	24.5864	7.4	24.4192	7.5
23	11.38	23.2296	8.7	23.2109	8.7
28	7.95	24.2383	7.7	24.1798	7.8
27	-	23.8798	8.1	23.9441	8.0
29	7.17-7.05	24.5861	7.4	24.7363	7.2
30	6.82-6.76	25.4045	6.5	24.5438	7.4
39	3.89-3.70	28.1858	3.8	25.3911	6.5
38	3.89-3.70	28.1533	3.8	28.1066	3.8
37	3.89-3.70	27.7777	4.2	28.126	3.8
32	3.89-3.70	28.0180	3.9	27.7053	4.2
31	3.89-3.70	28.0352	3.9	27.9863	3.9
33	3.89-3.70	27.6460	4.3	27.9857	3.9
25	7.17-7.05	24.6049	7.3	27.6808	4.3
36	3.89-3.70	28.3043	3.6	28.0531	3.9
34	3.89-3.70	28.0007	3.9	28.4023	3.5
35	3.89-3.70	27.8828	4.1	28.0407	3.9
40	2.35	29.2756	2.7	29.2027	2.7

41	2.35	29.7639	2.2	29.2743	2.6
42	2.35	29.4015	2.5	29.9443	2.0

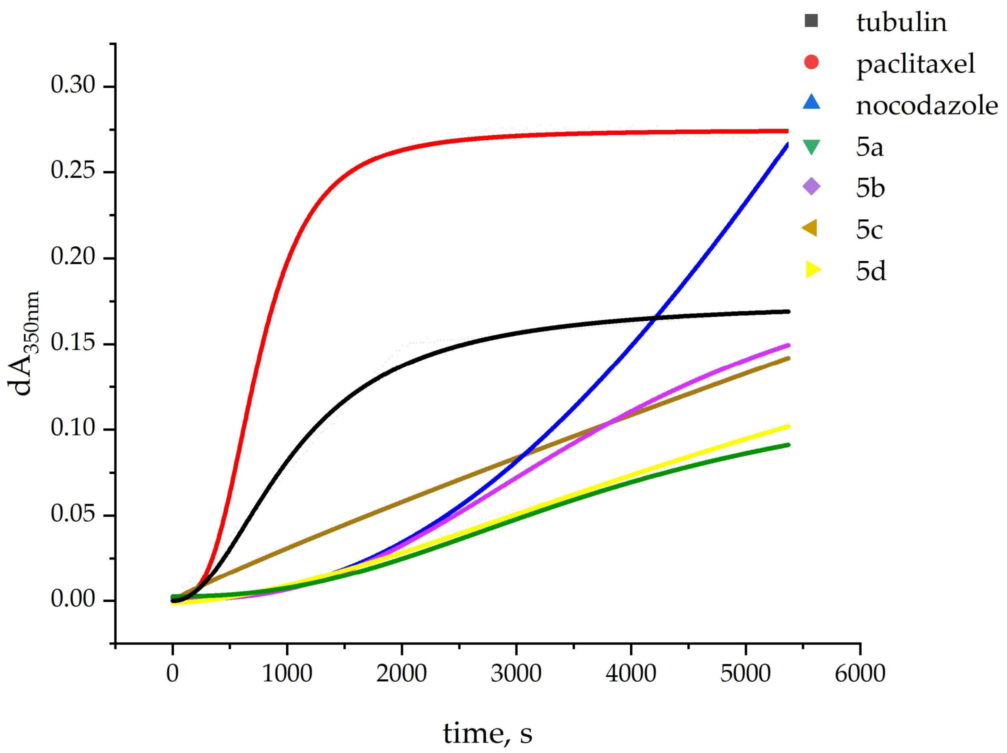

Figure S11. Tubulin (final 54 μM) in presence of PB-GTP buffer, 10 μM paclitaxel, 10 μM nocodazole and 10 μM of compounds **5a-d**. Reaction was conducted in a thermostated spec-trophotometric chamber at 37º C for 90 min. Turbidity was measured at 350 nm every 30 sec. Data for compounds **5a** and **5b** from [37].
